# Lesion Distribution in the Metacarpophalangeal and Metatarsophalangeal Region of 341 Horses Using Standing Magnetic Resonance Imaging

**DOI:** 10.3390/ani14131866

**Published:** 2024-06-25

**Authors:** Stefano Schiavo, Francesca Beccati, Rachel Pokora, Szu Ting Lin, Rebecca C. Milmine, Lars Bak, Vanessa G. Peter, Rachel C. Murray

**Affiliations:** 1 Rossdales Veterinary Surgeons, Cotton End Rd, Exning, Newmarket CB8 7NN, UK; rachel.pokora@rossdales.com (R.P.); vanessa.peter@rossdales.com (V.G.P.); 2 Sports Horse Research Centre, Department of Veterinary Medicine, University of Perugia, 06123 Perugia, Italy; francesca.beccati@unipg.it; 3 Department of Veterinary Medicine, University of Cambridge, Madingley Rd, Cambridge CB3 0ES, UK; linstjulie@outlook.com; 4 Dubai Equine Hospital, Dubai P.O. Box 9373, United Arab Emirates; rebecca.milmine@dubaiequine.ae; 5 Høejgård Equine Hospital, 8270 Højbjerg, Denmark; lb@eqvet.dk

**Keywords:** equine, MRI, fetlock, sport horse

## Abstract

**Simple Summary:**

Pathologies in the metacarpophalangeal (MCP)/metatarsophalangeal (MTP) region are a frequent cause of lameness in mature sport horses, and magnetic resonance imaging (MRI) of these regions is increasingly being acquired. The aims of this study were to describe ranges of abnormalities identified on standing MRI of the MCP/MTP region and compare patterns of abnormalities between forelimbs, hindlimbs, and different sports disciplines. MRI reports of 341 horses with lameness localised to the MCP/MTP region were reviewed. Joint and bone injuries were frequently identified with subchondral bone plate pathology, sclerosis and hyperintense signal on STIR sequences often reported. Medial pathology was more commonly identified in the forelimbs and lateral pathology in the hindlimbs, which potentially reflect differences in loading patterns. Significant differences in MRI findings were found between horses doing different sports activities, with reports from racing and endurance horses showing more bone pathology, while dressage and show jumping horses were frequently reported to have soft tissue injuries. Being aware of the type of abnormalities associated with particular sports could be useful for planning image acquisition and when interpreting MRI findings. Further investigation into associations between different types of pathology and particular clinical presentations, blocking patterns, and performance outcomes would be interesting.

**Abstract:**

Pain localised to the metacarpophalangeal (MCP) and metatarsophalangeal (MTP) region represents a frequent cause of lameness in sport horses, and standing magnetic resonance imaging (MRI) of these regions is increasingly being acquired. This multicentre retrospective study describes the ranges of abnormalities identified on standing MRI of the MCP/MTP region and compares patterns of abnormalities between forelimbs, hindlimbs and different sports disciplines. In total, 341 MRI reports were reviewed. Subchondral bone plate irregularities, condylar and proximal phalanx pathologies were frequently identified with subchondral bone defect, sclerosis and increased intensity on STIR images often described. Medial pathology was frequently identified in the forelimbs, and more lateral pathology was reported in the hindlimbs, which could potentially reflect differences in the loading patterns. Significant differences in MRI findings were found between different sports activities, with MCP/MTP bone pathology occurring more frequently in MRI reports from race and endurance horses and MCP/MTP soft tissue injuries being reported more frequently in dressage and show-jumping horses, particularly in the suspensory apparatus, including the distal sesamoidean ligaments. The findings of this study identify patterns of MCP/MTP abnormalities detected using standing MRI, with differences seen between forelimbs and hindlimbs and between different sports disciplines.

## 1. Introduction

Pain and lameness localised to the metacarpophalangeal (MCP) and metatarsophalangeal (MTP) region are a frequent cause of lameness in mature sports horses of all types [1]. The equine MCP and MTP joints are commonly affected by synovitis and/or osteoarthritis, and soft tissue pathology, including desmopathy and tendinopathy, have been described [1,2,3,4,5]. Diagnosis of specific injuries in this location with conventional imaging, such as radiography and ultrasonography, can be challenging [1,5,6,7,8]. The normal appearance of the equine MCP and MTP region on high- and low-field Magnetic Resonance Imaging (MRI) has been described in the previous literature [9,10].

High-field MRI has been recognised to provide information that is complementary to radiography, ultrasonography, and nuclear scintigraphy and allows for a comprehensive evaluation of all structures in the MCP and MTP regions [5]. Although standing low-field MRI is increasingly being used for the evaluation of these areas, to the author’s knowledge, data regarding MRI features in a large series of horses performing in different sports disciplines is limited [11]. The capacity of high- and low-field MRI of the MCP and MTP region to evaluate bone and articular pathology, including cartilage change, is recognised [1,7,12,13,14,15,16,17,18,19,20]. In addition, MRI is reported to be of value for the detection of soft tissue pathology in the fetlock region, including tendon and ligament pathology in horses, where this may not have been identified or fully evaluated using conventional imaging [21,22,23,24,25].

Horses used for different sports activities experience different loading patterns, and the injury risks appear to vary between sports [26,27,28]. It is possible that horses of different sports disciplines could develop a different balance of abnormalities in the MCP and MTP region, which could be detected using MRI. Being aware of the type of abnormalities associated with particular sports could be useful in the interpretation of imaging findings. Therefore, investigating the range of injury detected in horses undergoing MRI of the fetlock region could be useful in understanding patterns of injury associated with different sports and comparing these between the MCP and MTP regions.

Loading patterns experienced by the forelimbs and hindlimbs vary, related to the differences in gait between the fore- and hindlimbs, with forelimbs experiencing greater loading [29,30,31,32,33,34] than the hindlimbs and more compressive strain on the medial than lateral aspect [35,36,37,38,39,40]. Therefore, it can be expected that the patterns of abnormalities detected between the fore and hindlimbs detected on MRI may be different.

This study aimed to describe the range of abnormalities detected in horses undertaking different sports with lameness localised to the MCP and MTP region, undergoing standing low-field MRI examination. It was hypothesised that (1) the range of abnormalities detected in the MCP region would be different from the range of abnormalities detected in the MTP region and (2) horses of different sports disciplines would have a different pattern of MRI findings.

## 2. Materials and Methods

### 2.1. Inclusion Criteria

The study was multicenter retrospective in design and no approval by an ethical committee was required. The study evaluated MRI reports from horses undergoing standing low-field MRI using a 0.27 Tesla MRI system (Hallmarq Veterinary Imaging, Guildford, Surrey, UK) located at Rossdales Equine Hospital and Diagnostic Center (UK), Dubai Equine Hospital (UAE), and Høejgård Equine Hospital (Denmark) between January 2019 and 2022. Horses had undergone MRIs based on the judgement of the clinician concerned.

Prior to MRI examinations, patients had undergone a comprehensive lameness examination, radiography, and/or ultrasonography of the MCP/MTP region. The MRIs were performed to determine diagnosis and guide management. Reports were included in this study if the lameness had been localised to the MCP or MTP region using intra-articular and/or perineural (abaxial sesamoid and/or distal palmar/plantar and palmar metacarpal/plantar metatarsal) diagnostic analgesia or strong clinical evidence in cases where the risk of diagnostic analgesia was considered too high. Reports were excluded if horses were not lame or if the lameness had been localised to a different area. If limbs underwent an MRI scan more than once, only the first examination was included in this study. All images had been interpreted by one of two specialists: one diplomate ACVS, associate ECVDI, and RCVS specialist, and one diplomate ECVDI and RCVS specialist.

Information on horse signalment and sports discipline was obtained from the MRI report or established from the clinical records. The recorded MRI features were obtained from a report by a single trained researcher (advanced diagnostic imaging intern). Where there were queries about any feature described in the report, the MR images for the case were assessed by the original interpreter to confirm the finding reported.

The findings recorded included the structure affected (bone, joint or soft tissue), location and type of MRI abnormality identified, listed in Supplementary File S1.

### 2.2. Statistical Analysis

Descriptive statistics were performed to investigate the patterns of abnormalities reported. The relative proportions of each feature were compared between forelimbs and hindlimbs using a Chi-squared or Fisher’s exact test, as appropriate. Differences in the relative proportions of each feature in limbs from horses of different sports disciplines (flat-racing, endurance, pleasure riding, show-jumping, dressage and eventing) were investigated within each region using a Chi-squared or Fisher’s exact test, as appropriate. Residual analysis was performed to localise the origin of the significant difference. The significance level was set at *p* < 0.05. Abnormalities detected with a frequency of equal or less than 10 were not considered for the inferential statistics to avoid bias related to the low prevalence of the feature. Statistical analyses were performed using JASP (JASP Team 2022, version 0.16.4, The Netherlands) and R Studio (R Core Team 2022, R Foundation for Statistical Computing).

## 3. Results

### 3.1. Sample Population

The inclusion criteria were met by reports of 341 horses: 94 mares (27.6%), 198 geldings (58%), and 49 stallions (14.4%). The median age was 9 years (range 2–19 years). A total of 259 forelimbs (125 left; 134 right) and 82 hindlimbs (39 left; 43 right) were included in the study. The included forelimbs were from 102 Warmbloods/Warmblood cross, 74 Thoroughbreds/Thoroughbred cross, 44 Arabians/Arabian cross, 24 Ponies, 9 Irish Sport Horse, 2 Cobs, 2 Spanish, one Quarter horse, and one Icelandic horse. Hindlimbs were from 38 Warmbloods, 19 Thoroughbreds/Thoroughbred cross, 11 Arabians/Arabian cross, 6 ponies, 5 Irish Sport Horse, one Cob, one British Sport Horse, and one Irish Draught.

Of the forelimbs included, 71 were from horses performing in flat racing, 67 from dressage, 44 from endurance, 35 from show jumping, 22 from eventing, and 20 from pleasure riding. Of the hindlimbs reported, 30 were from dressage horses, 17 from flat racing, 12 from show jumping, 11 from endurance, 7 from pleasure riding, and 5 from eventing.

MRI sequences used included T1 and T2*-weighted gradient echo (GRE) FAST, T2 fast spin echo (FSE) FAST or SUPERFAST or motion insensitive and short tau inversion recovery (STIR) FSE FAST, motion insensitive and/or SUPERFAST. Images had been acquired in dorsal, transverse and sagittal planes with artefacts frequently reported, including motion, flow, and magic angle artefacts. Dorsal sequences had been acquired with orientation parallel to the long axis of the proximal phalanx and/or diaphysis of the third MC/MT bone. Transverse sequences were oriented perpendicular to the suspensory branches and proximal third of the proximal phalanx long axis and included the level of the distal sesamoidean ligaments distally and distal aspect of the suspensory branches proximally, with additional proximal and distal sequences acquired in cases in which detected bone injury or soft tissue pathology extended beyond the original region of interest.

### 3.2. MRI Findings

Distribution and type of abnormalities of the MCP/MTP regions overall are shown in Table 1a (osseous pathology), Table 1b (joint pathology) and Table 1c (soft tissue pathology).

#### 3.2.1. Osseous Pathology

Proximal phalanx cortical pathology was most frequently reported on the dorsoproximal aspect (n = 92; 26.9%), with bone margin irregularity (n = 85; 24.9%) and endosteal abnormalities (n = 104; 30.5%) being the most frequent. Trabecular bone pathology of the proximal phalanx was detected at a similar location, primarily dorsoproximal (n = 84; 24.6%) and medial (n = 54; 15.8%), with sclerosis detected in 47 of the total number of limbs (13.8%). Hyperintensity on STIR images was more frequently reported in the proximal phalanx’s trabecular bone (n = 79; 23.2%) than in cortical bone (n = 57; 16.7%).

Irregularities of the trabecular bone of the distal third MC/MT condyles were frequently described on the dorsal (n = 159; 46.6%) and palmar/plantar aspects (n = 140; 41.1%). The trabecular bone of the medial and lateral condyle of the third MC/MT bone was identified as a common location of abnormalities, including sclerosis (n = 176; 51.6%), hyperintensity on STIR images (n = 168; 49.3%) and dilation of the vascular channels (n = 114; 33.4%) at the level of the distal MC/MT epiphysis. Cortical bone abnormalities were less frequently reported, affecting the dorsal (n = 62; 26.9%) and palmar/plantar aspects (n = 51; 15%).

The proximal sesamoid bones (PSB) were most frequently reported to have abnormalities affecting the abaxial aspect of the medial (Med) (n = 98; 28.7%) and lateral (Lat) (n = 77; 22.6%) PSB.

#### 3.2.2. Joint Pathology

Frequently described MRI findings were joint effusion (n = 245; 71.9%), osteophyte formation (n = 203; 59.5%), as well as chronic synovitis, which was represented by joint distension and synovial proliferation (n = 219; 64.2%). Abnormalities affecting the subchondral bone plate of the MCP and MTP joint were frequently identified, including subchondral bone irregularity (n = 187/341; 54.8%), thickening/sclerosis (n = 258/341; 75.7%) and increased intensity on STIR images (n = 271; 79.5%).

**Figure 1:**
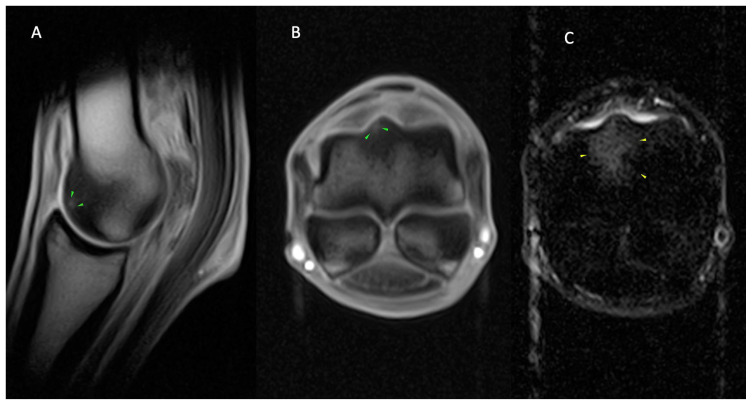
Lateral is to the left of the transverse images (**B**,**C**). From left, sagittal (**A**) and transverse T1 GRE (**B**) and transverse STIR FSE (**C**) images of the right MCP region of a horse with osseous and joint pathology. Well-defined, focal T1 GRE hyperintensity at the dorsolateral articular margin of the sagittal ridge (green arrows; (**A**,**B**)), which is surrounded by T1 GRE hypointense (**A**,**B**) and STIR FSE hyperintense signal (yellow arrows; (**B**)) of the dorsal sagittal ridge and adjacent dorsal half trabecular bone. The findings are consistent with the focal area of subchondral bone injury (green arrows; (**A**,**B**)) on the dorsolateral sagittal ridge, surrounded by a larger area of fluid signal ((**A**–**C**) (yellow arrows)).

Pathology identified on the dorsal aspect of the MCP/MTP joint was frequently identified both involving the dorsal aspect of the proximal phalanx (n = 252; 73.9%) and the dorsodistal aspect of the third MC and MT bone (n = 249; 73%). Abnormality at the level of the proximal phalanx sagittal groove was identified in 153 of the total number of limbs (44.9%). Third, MC/MT sagittal ridge joint pathology was also often described (n = 181; 53.1%). Reports of lesions affecting the subchondral bone plate of the proximal phalanx medial glenoid (n = 146; 42.8%) and medial condyle of the third MC/MT bone (n = 220; 64.5%) were more frequently reported than lateral (n = 72; 21.1% and n = 128; 37.5%, respectively) (Figure 1).

#### 3.2.3. Soft Tissue Pathology

**Figure 2:**
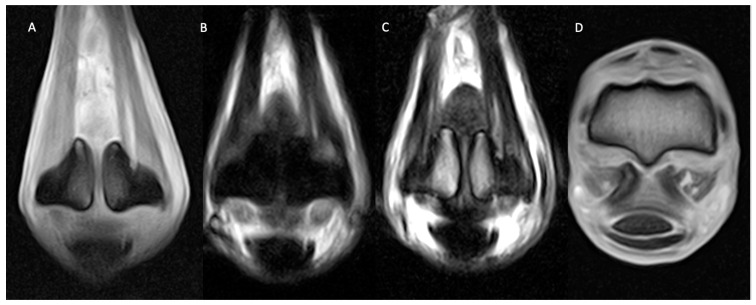
Lateral is to the left of the dorsal (**A**–**C**) and transverse (**D**) images. From left, dorsal T1 GRE (**A**), STIR FSE (**B**), T2 FSE (**C**), and transverse T1 GRE (**D**) images of the right MCP region of a horse with soft tissue and osseous pathology. There are well-defined, extensive, longitudinal injuries (hyperintense in all sequences) suggestive of active desmopathy of both suspensory branches (medial > lateral) with mild enlargement of the medial suspensory branch and moderate medial homogeneous periligamentous soft tissue swelling. Moderate abaxial bone remodelling and sclerosis of both proximal sesamoid bones are present with focal, well-defined, triangular-shaped STIR FSE hyperintense area (**B**) in the bone ligament interface, consistent with active medial enthesopathy.

Soft tissue injury of the medial and lateral branches of the suspensory ligament was frequently identified at the level of the branch (medial: n = 136; 39.9%; lateral: n = 91; 26.7%) rather than the distal attachment. Reports of lesions affecting the margin/periphery, with the axial aspect commonly affected, and the central aspect of the branch were common. The most frequent suspensory pathology types were increased STIR/T2 FSE hyperintensity (Med SB—n = 119, 34.9%; Lat SB—n = 92, 27%) or enlargement (Med SB—n = 73, 21.4%; Lat SB—n = 48, 14.1%), with splits (Med SB—n = 35, 10.3%; Lat SB—n = 23, 6.8%) and defects (Med SB—n = 67, 19.7%; Lat SB—n = 42, 12.3%) less frequently reported (Figure 2).

**Figure 3:**
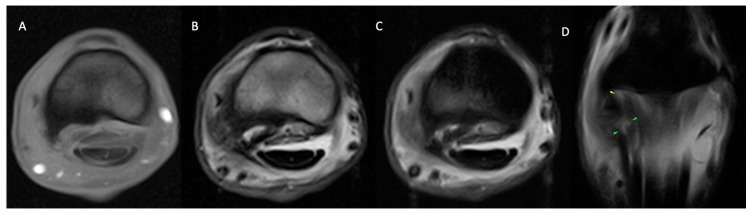
Lateral is on the left of the transverse (**A**–**C**) and dorsal (**D**) images. From left, transverse T1 GRE (**A**), T2 FSE (**B**), STIR FSE (**C**) and dorsal STIR FSE (**D**) images of the right MCP region of a horse with soft tissue and osseous pathology. Marked enlargement and prominent linear hyperintensity (in all sequences) of the lateral oblique distal sesamoidean ligament. The abnormal ligament is bordered on its lateral aspect by a large T1 GRE hypointense osseous spur (**A**) and marked T2 FSE heterogeneous periligamentous soft tissue swelling (**B**) in the region of the lateral aspect of the proximal digital annular ligament and lateral collateral sesamoidean ligament. T1 GRE (**A**)/T2 FSE (**B**) hypointense and STIR FSE (**C**) hyperintense signal in the palmarolateral half trabecular bone of the proximal phalanx. The findings are consistent with marked, active desmopathy and enthesopathy of the lateral oblique distal sesamoidean ligament +/− desmitis of the proximal digital annular ligament and lateral collateral sesamoidean ligament with associated marked bone marrow lesion (oedema-like) in the palmarolateral proximal phalanx.

A total of fifty-six limbs (16.4%) had abnormalities affecting the body of the medial oblique distal sesamoidean ligament, and 41 (12%) affected the body of the lateral oblique distal sesamoidean ligament. The type of lesion most frequently described had hyperintensity on STIR and/or T2 FSE images in the medial (n = 50; 14.7%) and lateral (n = 37; 10.9%) oblique distal sesamoidean ligaments (Figure 3).

Abnormalities of the lateral collateral ligament (LCL) of the MCP/MTP joint were uncommon but more frequently described than lesions affecting the medial collateral ligament (MCL) of the MCP/MTP joint with enlargement (LCL—n = 25, 7.3%; MCL—n = 4, 1.2%) and STIR/T2 FSE hyperintensity (LCL—n = 28, 8.2%; MCL—n = 8, 2.4%) representing the more commonly described pathologies.

The most frequently reported pathology affecting the digital flexor tendon sheath (DFTS) was distension of the synovial recess (n = 26, 7.6%) with mixed signal intensity, suggestive of synovial proliferation and chronic tenosynovitis (n = 20, 5.9%). Reports of lesions affecting the superficial and deep digital flexor tendons, palmar/plantar annular ligament, manica flexoria, straight distal sesamoidean ligament, intersesamoidean ligament, medial and lateral collateral sesamoidean ligament were relatively uncommon (Table 1c).

### 3.3. Comparison between Metacarpophalangeal and Metatarsophalangeal Regions

Table 2a–c, listed in Supplementary File S2, compares the lesion distribution between the MCP and MTP regions.

For pathology of the MCP/MTP joint, the medial condyle of the third MC/MT bone (*p* = <0.001) and medial aspect of the proximal phalanx (*p* = 0.004) were significantly more likely to be affected in the MCP than MTP region. A subchondral bone defect (*p* = <0.001), distension (*p* = 0.004) or chronic synovitis (*p* = 0.028) of the MCP/MTP joint were significantly more frequently detected in hindlimbs than forelimbs.

The cortical (*p* = 0.019) and trabecular (*p* = 0.001) bone pathologies of the third MC bone were significantly more likely to have the medial condyle affected than the third MT bone. Dilation of the vascular channels at the level of the distal condyles of the third MC/MT bone was reported significantly more frequently in the forelimbs than in hindlimbs (*p* = 0.005); the medial aspect of the cortical bone of the proximal phalanx in hindlimbs was more likely to be affected (*p* = 0.019) and to have endosteal (*p* = 0.028) irregularity (*p* = 0.012) than in forelimbs.

For the proximal sesamoid bones, forelimbs were more likely to have abnormalities identified in the medial proximal sesamoid bone (*p* = 0.005), with a greater likelihood of sclerosis (*p* = 0.004) than in hindlimbs. Reports from hindlimbs were more likely to include soft tissue injuries at the abaxial aspect of the medial (*p* = 0.022) and lateral (*p* = 0.045) suspensory branch, lateral collateral ligament of the MTP joint (*p* = 0.011), straight distal sesamoidean ligament (*p* = 0.047), axial (*p* = 0.034) and plantar (*p* = 0.049) aspect of the lateral oblique distal sesamoidean ligament, and superficial digital flexor tendon (*p* = 0.012) than in the forelimbs.

### 3.4. Comparison between Sports Disciplines

A comparison of lesion distribution between horses performing different sports activities is shown in Table 3a–c and listed in Supplementary File S3.

#### 3.4.1. Flat Racing

**Figure 4:**
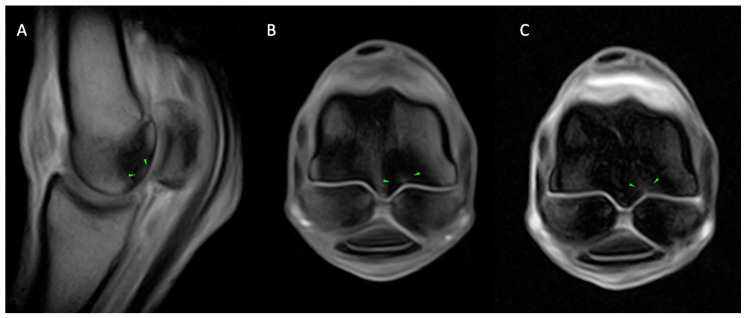
The lateral is to the right of the transverse images (**B**,**C**). From left, sagittal (**A**) and transverse T1 GRE (**B**), transverse T2* GRE (**C**) images of the left MCP region of a horse with a lateral parasagittal groove subchondral bone lesion. A small, focal and ill-defined area of moderate hyperintense signal in all sequences along the palmar aspect of the lateral parasagittal groove (green arrows; (**A**–**C**)) surrounded by moderate T1 hypointense signal (**A**,**B**) and mixed T2* GRE signal intensity with ill-defined fat-water (phase) cancellation artefact (**C**). Moderate intermediate T1 (**A**,**B**) and T2* GRE signal intensity (**C**) in the dorsal and palmar half of the medial condyle and dorsal half of the sagittal ridge.

**Figure 5:**
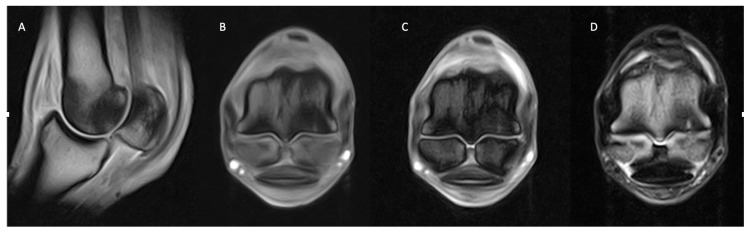
Lateral is to the left of the transverse images (**A**–**C**). From left, sagittal (**A**) and transverse T1 GRE, (**B**) transverse T2* GRE (**C**) and T2 FSE (**D**) images of the right MCP region of a horse with active bone and joint pathology. Well-defined T1 GRE and T2 FSE hypointensity and T2* GRE fat-water (phase) cancellation artefact is present in the palmar half of the medial third metacarpal condyle with a triangular to the linear area of marked hyperintensity present in all sequences, along the palmarodistal subchondral bone surface of the medial condyle.

Flat racehorses were overrepresented in reports of bone injury of the distal third MC/MT cortical and trabecular bone, including cortical bone abnormalities, condylar hypervascularity (*p* < 0.001), STIR hyperintensity (*p* < 0.001) and sclerosis (*p* = 0.004). Flat racehorses were significantly overrepresented in reports of lesions at the level of the dorsal (*p* = 0.011) and palmar/plantar (*p* < 0.001) distal condyles of the third MC/MT bone and proximal sesamoid bones, and, in particular, sclerosis of the medial proximal sesamoid bone (*p* < 0.001). Flat racehorses were over-represented in MCP/MTP joint abnormalities. Frequently reported lesions were described at the level of the palmar/plantar (*p* < 0.001) and lateral aspects of the third MC/MT bone (*p* < 0.001) and medial (*p* < 0.001) and lateral (*p* < 0.001) parasagittal groove of the proximal phalanx. Pathologies affecting the MCP/MTP joint frequently identified in flat racehorses were fissures (*p* < 0.001) and palmar/plantar osteochondral disease (*p* < 0.001) (Figure 4 and Figure 5).

Soft tissue injuries were significantly underrepresented in flat race-horses compared to other disciplines, specifically pathologies of the medial (*p* < 0.001) and lateral (*p* = 0.004) branches of the suspensory ligament and medial (*p* < 0.001) and lateral (*p* < 0.001) oblique distal sesamoidean ligaments.

#### 3.4.2. Dressage

MCP/MTP joint abnormalities were overrepresented or underrepresented at different locations of the articular surface in dressage horses compared to other sports disciplines. Commonly described abnormalities were reported at the level of the sagittal groove of the proximal phalanx (*p* < 0.001). Osteophytosis affecting the MCP/MTP joint was frequently identified in dressage horses (*p* < 0.001), whilst fissure pathologies at the level of the subchondral bone plate were significantly underrepresented (*p* < 0.001).

Specific soft tissue injuries were overrepresented in dressage horses compared to other sports disciplines. Significantly overrepresented injuries included chronic tenosynovitis of the digital flexor tendon sheath (*p* = 0.041), pathologies of the lateral branch of the suspensory ligament (*p* = 0.004), straight distal sesamoidean ligament (*p* = 0.009) and both medial (*p* = 0.001) and lateral (*p* < 0.001) oblique distal sesamoidean ligaments.

Dressage horses were significantly underrepresented in reports of bone injuries, specifically cortical and trabecular bone lesions at the level of the palmar/plantar and lateral condyle of the distal third MC/MT bone (*p* < 0.001). Dilation of the vascular channels at the level of the distal MC/MT condyles was also infrequently described (*p* < 0.001).

#### 3.4.3. Endurance

**Figure 6:**
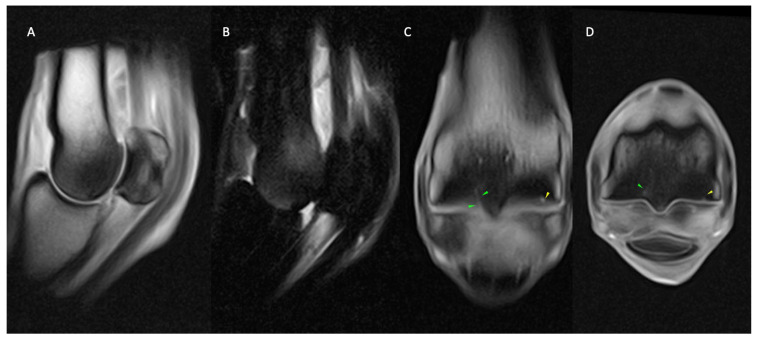
Lateral is to the left of the transverse (**D**) and dorsal (**C**) images. From left, sagittal T1 GRE (**A**) and STIR FSE (**B**), dorsal (**C**) and transverse T1 GRE (**D**) images of the right MCP region of a horse with osseous and fissure pathology. Well-defined, short, linear, T1 GRE hyperintensity extending from the palmar aspect of the lateral parasagittal groove (green arrows; (**C**,**D**)), surrounded by a large area of mixed T1 GRE hypointense (**A**,**C**,**D**) and STIR FSE hyperintense (**B**) signal extending throughout both metacarpal condyles. Focal, shallow T1 hyperintense subchondral outline irregularity along the palmar aspect of the medial metacarpal condyle (yellow arrows; (**C**,**D**)). The findings are consistent with fissure pathology of the palmar aspect of the lateral parasagittal groove (green arrows; (**C**,**D**)) and medial condylar focal area of subchondral bone resorption (yellow arrows; (**C**,**D**)) with marked, generalised condylar bone marrow lesion (oedema like) (**B**) and sclerosis (**A**,**C**,**D**) which are more evident in the lateral condyle.

Endurance horses were relatively overrepresented in reports of both bone and soft tissue injuries. Trabecular bone injury at the level of the palmar/plantar aspect of the third MC/MT bone was significantly overrepresented (*p* < 0.001, including subchondral bone irregularity (*p* = 0.002), fissures (*p* < 0.001), osteophyte formation (*p* < 0.001), and STIR hyperintensity at the level of the third MC/MT bone (*p* < 0.001) (Figure 6).

Cortical lesions at the level of the third MC/MT bone were underrepresented (*p* < 0.001) compared to other sport disciplines whilst cortical (*p* = 0.037), and in particular endosteal pathology (*p* = 0.02) affecting the dorsal aspect of the proximal phalanx was overrepresented in endurance horses, in particular bone margin irregularity (*p* = 0.001). Lesions involving the abaxial aspect of the lateral proximal sesamoid bone were relatively overrepresented in endurance horses (*p* = 0.013). Bone margin irregularity of both the medial (*p* = 0.006) and lateral (*p* < 0.001) proximal sesamoid bone and medial proximal sesamoid bone STIR hyperintensity (*p* = 0.009) were also overrepresented in endurance horses. Specific soft tissue injuries were frequently identified in endurance horses. Specifically, lesions at the level of the medial suspensory branch (*p* < 0.001) were significantly overrepresented in this sport discipline, with damage often identified at the level of the margin (*p* < 0.001) and, in particular, axially (*p* < 0.001), rather than the central aspect of the ligament. Medial suspensory branch defect (*p* < 0.001), split (*p* = 0.002), and STIR/T2 FSE hyperintensity (*p* < 0.001) were the most commonly described and overrepresented lesions.

#### 3.4.4. Show Jumping

Show jumpers were overrepresented in reports of pathology affecting the palmar/plantar aspect of the proximal phalanx articular surface (*p* = 0.003). Thickening of the synovial capsule (capsulitis) was frequently identified in show-jumping horses (*p* < 0.001). On the contrary, horses belonging to this sports discipline were significantly underrepresented in reports of trabecular bone lesions at the level of the palmar/plantar aspect of the distal third MC/MT bone (*p* < 0.001).

Abnormalities of the medial branch of the suspensory ligament and medial oblique distal sesamoidean ligament were significantly overrepresented in show-jumping horses (*p* < 0.001). Frequently reported lesions of the medial branch of the suspensory ligament were identified at the level of the insertion to the medial proximal sesamoid bone (*p* < 0.001); commonly described abnormalities of the medial oblique distal sesamoidean ligament were reported at the level of the axial aspect of the ligament (*p* < 0.001). Enlargement of the medial branch of the suspensory ligament (*p* = 0.032) and margin irregularity of the medial oblique distal sesamoidean ligament (*p* < 0.001) were significantly overrepresented in show jumpers.

#### 3.4.5. Eventing

Soft tissue injuries were relatively uncommonly reported in eventing horses. However, abnormalities of the deep digital flexor tendon, distal to the proximal sesamoid bones (*p* = 0.003), and medial distal oblique sesamoid ligament (*p* = 0.001) were frequently identified. Event horses were relatively overrepresented for deep digital flexor margin irregularity (*p* = 0.021), thickening (*p* = 0.04), and adhesion formation with the surrounding soft tissue structures (*p* = 0.019). Similar to show jumping, event horses were underrepresented in reports of bone injury at the level of the third MC/MT bone, including lesions at the level of the cortical bone of the third MC/MT medial condyle (*p* < 0.001) and of STIR hyperintensity in the distal third MC/MT bone (*p* < 0.001) and lateral proximal sesamoid bone (*p* = 0.013). Lesions of the proximal phalanx sagittal groove were not described in the population of eventing horses included in this study, whereas capsulitis was frequently identified (*p* < 0.001).

#### 3.4.6. Pleasure Riding

In pleasure horses, reports of soft tissue injuries were commonly identified, while bone and MCP/MTP joint injuries were uncommon. In comparison to other sports disciplines, pleasure horses were significantly overrepresented in reports of pathologies of the lateral collateral ligament of the MCP/MTP joint (*p* < 0.001), palmar/plantar annular ligament (*p* < 0.001), lateral lobe of the deep digital flexor tendon (*p* = 0.019), superficial digital flexor tendon (*p* = 0.038) and lateral oblique distal sesamoid ligament (*p* < 0.001).

## 4. Discussion

This multicenter study describes the patterns of MRI abnormalities identified in horses with lameness localised to the MCP/MTP region. The results support our hypotheses, with differences in patterns of injury between forelimbs and hindlimbs and between horses doing different sports activities.

Comparison between metacarpophalangeal and metatarsophalangeal regions

In our study, reports of subchondral bone pathologies of the medial third MC bone and medial glenoid of the proximal phalanx were more frequently identified in the forelimbs than in the hindlimbs. Forelimbs were also more likely to have pathology reported in the medial proximal sesamoid bone, with a greater likelihood of sclerosis than in hindlimbs. This may relate to the greater loading on the medial aspect of the third MC than MT. It has previously been suggested that a high frequency of MCP joint pathology in forelimbs may reflect differences in loading between fore- and hindlimbs, with forelimbs experiencing greater loading (ca. 60%), particularly medially, and kinematic stress than the hindlimbs [29,30,31,32,33,34,35,36,37,38,39,40]. Increased loading in forelimbs, particularly in high-intensity training sports disciplines, could explain a biomechanical link with the medial MCP rather than MTP joint pathology detected in this study, as well as the higher prevalence in sports disciplines, including flat-racing, endurance, eventing, and show jumping compared to general purpose horses.

There is growing evidence of proximal phalanx subchondral bone pathology in sport horses, potentially secondary to chronic bone overload or stress injury [18,20,41,42]. In a similar pattern to medial condylar pathology, medial subchondral bone pathology of the proximal phalanx was relatively more common in forelimbs than hindlimbs, supporting relatively greater medial loading in forelimbs and potential stress-related injury [34]. Condylar and proximal phalanx bone adaptation has been shown to follow a distinct pattern in response to chronic stress overloading [1,7,11,19,43,44,45,46,47,48], particularly in horses undergoing high-intensity training, such as flat racing [2,3,14,16,49,50,51] and endurance horses [52,53]. During functional adaptation to exercise, bone modelling adds additional bone and bone remodelling repairs damaged bone [54]. Bone strength is highly dependent on the ability of the bone to dissipate the stress that leads to an increase in strain, as well as on the microstructural properties that prevent crack propagation [55]. High-intensity training may lead to bone microcracks or inappropriate remodelling and can potentially be prodromal to catastrophic injury [2,3,20,49,56].

Comparison between sports disciplines

When MRI findings were compared between different sports activities, significant differences in patterns of abnormalities were found. Dilation of the vascular channels of the distal third MC condyles occurred more frequently in flat racehorses in this study, supporting the findings of a recent study [57] which proposed a correlation between the increased mineralisation and the enlarged vascular channels in the metacarpal condyles as a result of bone remodelling and adaptive changes occurring at the beginning of a horse’s race-training career. The findings in our study population may reflect adaptation to increased loading and biomechanical stress at these locations, particularly in horses in race training.

In our population, flat racing horses were overrepresented in MRI reports where fissure pathology was identified. The prevalence of fissures in our study (43.2%) was similar to that of a previous study [58] in Thoroughbred forelimbs (40.3%) and higher than another study [18] in Thoroughbreds, which included pelvic limbs (35.8%). Flat racing horses were found to be underrepresented in reports for osteophytosis of the MCP/MTP joint. It is possible that this relates to the fact that racehorses typically commence their sports career at a young age. Consequently, they show a lower prevalence of degenerative processes that typically manifest with advancing age.

The proportion of flat racing horses with soft tissue injury was relatively low but higher than in a previous study [18]. In our study, the most commonly reported soft tissue injury was the body of the medial suspensory branch. Subclinical suspensory branch desmopathy has been described as an increasing concern in racehorses [59,60,61] and their early detection may be beneficial from a long-term career perspective.

Similar to the flat racing discipline, endurance horses were overrepresented in fissures. This may relate to the repetitive and cyclical nature of the loads in endurance horses and also associated with increasing speeds, particularly in endurance horses from the Middle East, as in our sample population, making their biomechanics more similar to racing horses in the location of fatigue damage. These findings are in line with a previous report which described racehorse-type fractures in endurance horses and speculated that they may be related to increasing speed [62]. Compared to a report from endurance horses trained in Italy, the prevalence of fissures was considerably higher in our study (40% vs. 0.3%) [52]. The same previous study [52] identified arthropathy as the second most common orthopaedic injury after high suspensory disease. Abnormalities of the medial suspensory ligament branch were the most common soft tissue injury we reported in endurance horses, concordant with other studies [52,63]. In contrast to this previous study, our study reports were from a range of locations, with a larger proportion from the Middle East, where horses are trained and compete on sand and on long desert tracks at moderate to high speeds (24 to 30 km/h) [64]. It is possible that differences in speed and ground conditions between horses trained and competing in the Middle East compared to Europe could influence differences in lesion distribution and incidence of pathology, with horses trained at higher speed, over sandy surfaces and for long distances, being at higher risk of fissure lesions.

In comparison to endurance and flat racehorses, show jumping and dressage horses were relatively underrepresented in reports for bone injury at the level of the third MC/MT bone, particularly at the palmar/plantar aspect of both condyles and overrepresented in subchondral bone lesions of the proximal phalanx. Dorsal and medial third MC/MT condylar pathology was frequently identified in MRI reports of show jumpers, although no significant prevalence was found compared to other sports disciplines. Recent research [65] has suggested that densification in the sagittal ridge and third MC condyles, observed in MRI and computed tomography (CT) scans of 31 non-lame Warmblood show jumpers, likely reflects an adaptive response to jumping exercise. This may reflect loading patterns at take-off or landing, where fetlock hyperextension and vertical loading increase with the height of the fence [66]. Resorptive lesions in the sagittal groove of the proximal phalanx have been associated with subchondral bone trauma and fissure pathologies in sport horses [20,48,67,68]. It seems likely that the differences in loading patterns between sports disciplines could be contributing to the difference in prevalence of these lesions. It was suggested [47] and supported by the results of our study that constant turning in jumping and rotational movements in dressage horses might influence forces between the sagittal ridge of the third MC/MT bone and the sagittal groove of the proximal phalanx.

Overall, a low frequency of soft tissue injuries was found in this study, which is in contrast with previous publications where soft tissue pathologies were identified more frequently than subchondral bone or articular cartilage injuries with standing low-field [11] and high-field MRI in horses with radiographically normal MCP/MTP joints [69]. It is possible that soft tissue injuries are often primarily diagnosed with conventional imaging, like ultrasonography, so either the horses may not proceed to MRI, or MRI is being used as an adjunct imaging tool to further characterise the lesion or to identify concurrent bone pathology. Similar to bone pathologies, it has been recognised how soft tissue injuries, particularly suspensory desmopathy, can be associated with hyperextension of the MCP/MTP joint [70,71]. During movement, the extension of the MCP/MTP joint results in strain on the entire suspensory apparatus, including the distal sesamoidean ligaments. The straight sesamoidean ligament stabilises the MCP/MTP joint in a sagittal direction, whilst the oblique distal sesamoidean ligaments are essential to limit rotation and collateral motion of the MCP/MTP joint when the limb is under weight-bearing [22]. As part of the suspensory apparatus, the distal sesamoidean ligaments prevent hyperextension of the MCP/MTP joint [71,72]. Repetitive extension of the MCP/MTP joint is exacerbated in some sports disciplines, such as show jumping and dressage [73]. This could explain why, despite soft tissue injury being relatively uncommonly reported in our study overall, injuries of the suspensory apparatus, including distal sesamoidean ligaments, were overrepresented in some sports disciplines.

Soft tissue injuries, in particular desmopathy of the straight and oblique distal sesamoidean ligaments, have been identified as a frequent finding in standing low- [5] and high-field MRI [72] of the MC/MT region. Similar to previous studies, we found pathology of the oblique distal sesamoidean ligaments more commonly reported than the straight distal sesamoidean ligament [21,74].

Medial desmopathy is more often reported than lateral in forelimbs [21,75,76], but may relate to the majority of reports in forelimbs, where we found, in accordance with previous publications [21,74], lateral oblique distal sesamoidean desmopathy is more frequently reported in hindlimbs than forelimbs. This may relate to differences in biomechanics between forelimbs and hindlimbs [75]. There is a tendency for hindlimbs to have higher frequencies of heel first contact than the forelimbs, especially during high-speed locomotion [34]; there is also the tendency for most horses to land on the lateral side of the foot, especially in hindlimbs [77] and for the lateral aspect of the hoof to sink into a soft surface. Repetitive strains on the plantarolateral soft tissue structures could be associated with plantarolateral hyperextension of the MTP could explain the greater frequency of occurrence in hindlimbs and on the lateral aspect of the MTP region. Suspensory branch desmopathy, straight distal sesamoidean ligament and superficial and deep digital flexor tendinopathy were often reported in hindlimbs with a tendency to identify lateral pathology more often than medial in the case of collateral ligaments of the MTP joint and oblique distal sesamoidean ligaments desmopathy.

This study has limitations, largely related to its retrospective design. Sample size calculation for the inferential statistic was not performed because of the need to calculate it for each feature included, which was not practical considering the high number of MRI findings considered and the clinical interest for each of them. However, inferential statistics were limited to MRI features, with a minimum of 10 cases detected, and appropriate statistical correction was applied to the tables to avoid bias. It is possible that the interpretation of the relative clinical significance of MRI findings could have varied between clinicians, but both interpreters had worked together closely, so large differences in opinions were not considered likely. The presence of multiple concurrent pathologies, which are potentially related, would be a frequent finding in our experience, and MRI has played a crucial role in enhancing awareness of these complex scenarios. The lack of a standardised diagnostic analgesia approach and clinical examination was also a limitation of this study because the clinical evaluation in the majority of the cases was done by a referring veterinarian surgeon. However, in all cases, the veterinarians involved had extensive experience in equine sports medicine and orthopaedics and had a close relationship with the team acquiring and interpreting the images.

## 5. Conclusions

Osseous and subchondral bone pathology were frequent MRI findings in our horse population, with different patterns of lesion distribution between forelimbs and hindlimbs. Medial pathology was more commonly identified in the forelimbs and lateral pathology in the hindlimbs, which potentially reflect differences in loading patterns. Significant differences in MRI findings were also found between sports activities, with reports from racing and endurance horses showing more bone pathology, while dressage and show jumping horses were more frequently reported to have soft tissue injuries. Being aware of the type of abnormalities associated with particular sports could be useful for targeting image acquisition and when interpreting MRI findings. Further investigations into associations between different types of pathology and particular clinical presentations, blocking patterns, and performance outcomes would be interesting.

## Figures and Tables

**Table 1:** Distribution and type of abnormalities in the metacarpophalangeal/metatarsophalangeal region of 341 limbs of horses undergoing standing MRI with number (N) and proportion (%) of limbs affected. (a) Distribution and pathology type affecting the bones of the metacarpophalangeal/metatarsophalangeal region with number (N) and proportion (%) of limbs affected. (b) Distribution and pathology type affecting the metacarpophalangeal/metatarsophalangeal joint and digital flexor tendon sheath lesions with number (N) and proportion (%) of limbs affected. (c) Distribution and pathology type affecting the soft tissues of the metacarpophalangeal/metatarsophalangeal region with number (N) and proportion (%) of limbs affected.

**(a)** **Anatomical Location** **Bone**				**MRI Features**	**Number (N = 341)** **and Proportion (%)** **of Limbs Affected**
**MC/T 3**	**Cortex**		Location	Dorsal	62 (26.9)
				Palmar/Plantar	51 (15)
				Axial (sagittal ridge)	37 (10.9)
				Medial	58 (17)
				Lateral	39 (11.4)
			Aspect	Periosteal	57 (16.7)
				Endosteal	74 (21.7)
				All	59 (17.3)
			Pathology type	Irregularity	30 (8.8)
				Sclerosis	55 (16.1)
				Fluid (hyperintensity)	50 (14.7)
	**Trabecular** **bone**		Location	Dorsal	159 (46.6)
				Palmar/Plantar	140 (41.1)
				Axial (sagittal ridge)	80 (23.5)
				Medial	181 (53.1)
				Lateral	114 (33.4)
			Pathology type	Margin irregularity	46 (13.5)
				Sclerosis	176 (51.6)
				Fluid (hyperintensity)	168 (49.3)
				Fracture incomplete	13 (3.8)
				Hypervascularisation	114 (33.4)
**P1**	**Cortex**		*Location*	Dorsal	92 (26.9)
				Palmar/Plantar	14 (4.1)
				Axial (sagittal groove)	59 (17.3)
				Medial	48 (14.1)
				Lateral	20 (5.9)
			*Aspect*	Periosteal	63 (18.5)
				Endosteal	104 (30.5)
				All	60 (17.6)
			*Pathology* *type*	Irregularity	85 (24.9)
				Sclerosis	43 (12.6)
				Fluid (hyperintensity)	57 (16.7)
	**Trabecular** **bone**		*Location*	Dorsal	84 (24.6)
				Palmar/Plantar	18 (5.3)
				Axial (sagittal groove)	44 (12.9)
				Medial	54 (15.8)
				Lateral	24 (7)
			*Pathology* *type*	Margin irregularity	45 (13.2)
				Sclerosis	47 (13.8)
				Fluid (hyperintensity)	79 (23.2)
PSB	Medial	Location		Axial	56 (16.4)
				Abaxial	98 (28.7)
				Proximal	63 (18.5)
				Distal	56 (16.4)
				Diffuse	55 (16.1)
		Pathology type		Irregularity	61 (17.9)
				Sclerosis	38 (11.1)
				Fluid (hyperintensity)	68 (19.9)
	Lateral	Location		Axial	48 (14.1)
				Abaxial	77 (22.6)
				Proximal	49 (14.4)
				Distal	44 (12.9)
				Diffuse	45 (13.2)
		Pathology type		Irregularity	42 (12.3)
				Sclerosis	28 (8.2)
				Fluid (hyperintensity)	67 (19.7)
**(b)** **Anatomical Location** **Joint**				**MRI Features**	**Number (N = 341)** **and Proportion (%)** **of Limbs Affected**
**MC/MTP**	Location			Dorsal P1	252 (73.9)
				Dorsal MC/T 3	249 (73)
				Palmar/Plantar P1	60 (17.6)
				Palmar/Plantar MC/T 3	160 (46.9)
				Proximal P1	262 (76.8)
				Distal MC/T 3	297 (87.1)
				Medial P1	146 (42.8)
				Medial MC/T 3	220 (64.5)
				Lateral P1	72 (21.1)
				Lateral MC/T 3	128 (37.5)
				Axial P1 (sagittal groove)	153 (44.9)
				Medial parasagittal groove	70 (20.5)
				Lateral parasagittal groove	69 (20.2)
				Axial MC/T3 (sagittal ridge)	181 (53.1)
	Pathology type			Subchondral bone Irregularity	187 (54.8)
				Sclerosis	258 (75.7)
				Fluid (hyperintensity)	271 (79.5)
				Cartilage irregularity/fibrillation	26 (7.6)
				Fissure	66 (19.4)
				Osteophyte	203 (59.5)
				POD	16 (4.7)
				Distension	245 (71.9)
				Chronic synovitis	219 (64.2)
				Capsulitis	75 (22)
**DFTS**	Pathology type			Distension	26 (7.6)
				Chronic synovitis	20 (5.9)
**(c)** **Anatomical Location** **Soft Tissue**				**MRI Features**	**Number (N = 341)** **and Proportion (%)** **of Limbs Affected**
**Med SB**	Location			Insertion	62 (18.2)
				Body	136 (39.9)
	Part of the branch affected			Margin/periphery	113 (33.1)
				Central/core	91 (26.7)
				Entire branch	23 (6.8)
	Area of the margin affected			Dorsal	24 (7)
				Palmar/plantar	42 (12.3)
				Axial	108 (31.7)
				Abaxial	29 (8.5)
	Pathology type			Defect	67 (19.7)
				Fluid-based hyperintensity	119 (34.9)
				Enlargement	73 (21.4)
**Lat SB**	Location			Insertion	50 (14.7)
				Body	91 (26.7)
	Part of the branch affected			Margin/periphery	73 (21.4)
				Central/core	70 (20.5)
				Entire branch	31 (9.1)
	Area of the margin affected			Dorsal	28 (8.2)
				Palmar/plantar	39 (11.4)
				Axial	74 (21.7)
				Abaxial	31 (9.1)
	Pathology type			Defect	42 (12.3)
				Fluid-based hyperintensity	92 (27)
				Enlargement	48 (14.1)
**MCL**	Location			Dorsal	8 (2.4)
				Palmar/plantar	7 (2.1)
				Axial	8 (2.4)
				Abaxial	8 (2.4)
				Entire ligament	8 (2.4)
	Level			Superficial (long)	8 (2.4)
				Deep (short)	9 (2.6)
				Origin	7 (2.1)
				Body	7 (2.1)
				Insertion	6 (1.8)
				Entire ligament	2 (0.6)
	Pathology type			Fluid-based hyperintensity	8 (2.4)
				Enlargement	4 (1.2)
**LCL**	Location			Dorsal	26 (7.6)
				Palmar/plantar	23 (6.8)
				Axial	30 (8.8)
				Abaxial	22 (6.5)
				Entire ligament	20 (5.9)
	Level			Superficial (long)	27 (7.9)
				Deep (short)	29 (8.5)
				Origin	22 (6.5)
				Body	20 (5.9)
				Insertion	14 (4.1)
				Entire ligament	10 (2.9)
	Pathology type			Fluid-based hyperintensity	28 (8.2)
				Enlargement	25 (7.3)
**SDSL**	Location			Dorsal	9 (2.6)
				Palmar/plantar	8 (2.4)
				Medial	11 (3.2)
				Lateral	7 (2.1)
				Entire ligament	6 (1.8)
	Level			Bone ligament interface	3 (0.9)
				Body	13 (3.8)
	Pathology type			Irregularity	8 (2.4)
				Enlargement	5 (1.5)
				Fluid-based hyperintensity	7 (2.1)
				Adhesions	5 (1.5)
**Med ODSL**	Location			Dorsal	26 (7.6)
				Palmar/plantar	29 (8.5)
				Axial	49 (14.4)
				Abaxial	25 (7.3)
				Entire ligament	25 (7.3)
	Level			Bone ligament interface	7 (2.1)
				Body	56 (16.4)
	Pathology type			Irregularity	33 (9.7)
				Enlargement	27 (7.9)
				Fluid-based hyperintensity	50 (14.7)
				Adhesions	4 (1.2)
**Lat ODSL**	Location			Dorsal	27 (7.9)
				Palmar/plantar	28 (8.2)
				Axial	40 (11.7)
				Abaxial	25 (7.3)
				Entire ligament	23 (6.8)
	Level			Bone ligament interface	4 (1.2)
				Body	41 (12)
	Pathology type			Irregularity	24 (7)
				Enlargement	30 (8.8)
				Fluid-based hyperintensity	37 (10.9)
				Adhesions	3 (0.9)
**DDFT**	Location			Dorsal	4 (1.2)
				Palmar/plantar	2 (0.6)
				Medial	1 (0.3)
				Lateral	4 (1.2)
	Level			Proximal to the PSB	5 (1.5)
				At the level of the PSB	3 (0.9)
				Distal to the PSB	3 (0.9)
				Body	3 (0.9)
	Pathology type			Irregularity	6 (1.8)
				Enlargement	1 (0.3)
				Fluid-based hyperintensity	2 (0.6)
				Adhesions	4 (1.2)
				Chronic fibrosis	4 (1.2)
**SDFT**	Location			Dorsal	8 (2.4)
				Palmar/plantar	3 (0.9)
				Medial	9 (2.6)
				Lateral	3 (0.9)
	Level			Proximal to the PSB	5 (1.5)
				At the level of the PSB	12 (3.5)
				Distal to the PSB	7 (2.1)
				Body	5 (1.5)
	Pathology type			Irregularity	14 (4.1)
				Enlargement	4 (1.2)
				Fluid-based hyperintensity	12 (3.5)
				Adhesions	7 (2.1)
				Chronic fibrosis	2 (0.6)

MCP j = metacarpophalangeal joint; MTP j = metatarsophalangeal joint; MC 3 = third metacarpal bone; MT 3 = third metatarsal bone; P1 = proximal phalanx; PSB = proximal sesamoid bone; POD = palmar/plantar osteochondral disease; DFTS = digital flexor tendon sheath; FL > HL = forelimbs overrepresented; HL > FL = hindlimbs overrepresented. Med SB = medial suspensory branch; Lat SB = lateral suspensory branch; MCL = medial collateral ligament of the metacarpophalangeal/metatarsophalangeal joint; LCL = lateral collateral ligament of the metacarpophalangeal/metatarsophalangeal joint; SDSL = straight distal sesamoidean ligament; MODSL = medial oblique distal sesamoidean ligament; LODSL = lateral oblique distal sesamoidean ligament; DDFT = deep digital flexor tendon; SDFT = superficial digital flexor tendon.

**Table 2:** MRI features from horses undergoing standing magnetic resonance imaging of the metacarpophalangeal/metatarsophalangeal region with number (N) and proportion (%) of forelimbs and hindlimbs affected. Features with significant differences in proportions between forelimbs and hindlimbs (Pearson’s Chi-squared test) are shown, with the overrepresented category in bold, using a significance level of *p* < 0.05. FL = forelimbs; HL = hindlimbs; MCP j = metacarpophalangeal joint; MTP j = metatarsophalangeal joint; MC 3 = third metacarpal bone; MT 3 = third metatarsal bone; P1 = proximal phalanx; PSB = proximal sesamoid bone; POD = palmar/plantar osteochondral disease; DFTS = digital flexor tendon sheath; Med SB = medial suspensory branch; Lat SB = lateral suspensory branch; MCL = medial collateral ligament of the metacarpophalangeal/metatarsophalangeal joint; LCL = lateral collateral ligament of the metacarpophalangeal/metatarsophalangeal joint; SDSL = straight distal sesamoidean ligament; MODSL = medial oblique distal sesamoidean ligament; LODSL = lateral oblique distal sesamoidean ligament; DDFT = deep digital flexor tendon; SDFT = superficial digital flexor tendon. (a) Distribution and pathology type of bone lesions affecting the forelimb and hindlimbs of the metacarpophalangeal/metatarsophalangeal region with number (N) and proportion (%) of forelimbs and hindlimbs affected. (b) Distribution and pathology type of metacarpophalangeal/metatarsophalangeal joint and digital flexor tendon sheath lesions in forelimbs and hindlimbs. (c) Distribution and pathology type affecting the soft tissues of the metacarpophalangeal/metatarsophalangeal region with number (N) and proportion (%) of forelimbs and hindlimbs affected.

**(a)** **Anatomical Location** **Bone**			**MRI Features**	**Number (N = 259) and Proportion (%) of** **FL Affected**	**Number (N = 82) and Proportion (%) of** **HL Affected**	***p* value**
**MC/T 3**	**Cortex**	Location	Medial	**51 (19.7)**	7 (8.5)	**0.019**
	**Trabecular** **bone**	Location	Axial (sagittal ridge)	**68 (26.3)**	12 (14.6)	**0.03**
			Medial	**151 (58.3)**	30 (36.6)	**0.001**
		Pathology type	Hypervascularisation	**97 (37.5)**	17 (20.7)	**0.005**
**P1**	**Cortex**	Location	Medial	30 (11.6)	**18 (22)**	**0.019**
		Aspect	Endosteal	71 (27.4)	**33 (40.2)**	**0.028**
		Pathology type	Irregularity	56 (21.6)	**29 (35.4)**	**0.012**
**PSB**	**Medial**	Location	Axial	**50 (19.3)**	6 (7.3)	**0.011**
			Abaxial	**82 (31.7)**	16 (19.5)	**0.034**
			Proximal	**56 (21.6)**	7 (8.5)	**0.008**
			Diffuse	**50 (19.3)**	5 (6.1)	**0.005**
		Pathology type	Sclerosis	**36 (13.9)**	2 (2.4)	**0.004**
**(b)** **Anatomical Location** **Joint**			**MRI Features**	**Number (N = 259) and Proportion (%) of** **FL Affected**	**Number (N = 82) and Proportion (%) of** **HL Affected**	***p* value**
**MC/MTP**	**Location**		Medial P1	**122 (47.1)**	24 (29.3)	**0.004**
			Medial MC/T 3	**182 (70.3)**	38 (46.3)	**<0.001**
	**Pathology** **type**		Subchondral bone defect	45 (28)	**17.4 (34.2)**	**<0.001**
			Distension	176 (68)	**69 (84.2)**	**0.004**
			Chronic synovitis	158 (61)	**61 (74.4)**	**0.028**
**DFTS**	**Pathology** **type**		Distension	14 (5.4)	**12 (14.6)**	**0.006**
			Chronic synovitis	10 (3.9)	**10 (12.2)**	**0.011**
**(c)** **Anatomical Location** **Soft Tissue**			**MRI Features**	**Number (N = 259) and Proportion (%) of** **FL Affected**	**Number (N = 82) and Proportion (%) of** **HL Affected**	***p* value**
**Med SB**	Area of the margin affected		Abaxial	17 (6.6)	**12 (14.6)**	**0.022**
	Pathology type		Enlargement	48 (18.5)	**25 (30.5)**	**0.021**
**Lat SB**	Area of the margin affected		Abaxial	19 (7.3)	**12 (14.6)**	**0.045**
	Pathology type		Enlargement	30 (11.6)	**18 (22)**	**0.006**
**MCL**	Location		Insertion	2 (0.8)	**4 (4.9)**	**0.047**
**LCL**	Location		Dorsal	14 (5.4)	**12 (14.6)**	**0.006**
			Palmar/plantar	11 (4.3)	**12 (14.6)**	**0.001**
			Axial	16 (6.2)	**14 (17.1)**	**0.002**
			Abaxial	10 (3.9)	**12 (14.6)**	**<0.001**
			Entire ligament	10 (3.9)	**10 (12.2)**	**0.011**
	Level		Superficial (long)	14 (5.4)	**13 (15.9)**	**0.002**
			Deep (short)	15 (5.8)	**14 (17.1)**	**0.001**
	Pathology type		Fluid-based hyperintensity	15 (5.8)	**13 (15.9)**	**0.004**
			Enlargement	13 (5.2)	**12 (14.6)**	**0.004**
**SDSL**	Location		Entire ligament	2 (0.8)	**4 (4.9)**	**0.047**
**Lat ODSL**	Location		Palmar/plantar	17 (6.6)	**11 (13.4)**	**0.049**
			Axial	25 (9.7)	**15 (18.3)**	**0.034**
**SDFT**	Level		Distal to the PSB	2 (0.8)	**5 (6.1)**	**0.012**

**Table 3:** MRI features from horses undergoing standing magnetic resonance imaging of the metacarpophalangeal/metatarsophalangeal region from horses with a history of undertaking different sports with a number (N) and proportion (%) of forelimbs and hindlimbs affected. Features with significant differences in proportions between different sports (Pearson’s Chi-squared test) are shown with overrepresented category in bold and underrepresented underlined. They were categorised using a significance level of *p* < 0.05. (a) Distribution and pathology type of bone lesions of the metacarpophalangeal/metatarsophalangeal region from horses with a history of undertaking different sports with number (N) and proportion (%) of forelimbs and hindlimbs affected. (b) Distribution and pathology type of metacarpophalangeal/metatarsophalangeal joint and digital flexor tendon sheath lesions from horses with a history of undertaking different sports with number (N) and proportion (%) of forelimbs and hindlimbs affected. (c) Distribution and pathology type affecting the soft tissues of the metacarpophalangeal/metatarsophalangeal region from horses with a history of undertaking different sports with number (N) and proportion (%) of forelimbs and hindlimbs affected.

**(a)** **Anatomical Location** **Bone**			**MRI Features**	**Dressage** **N = 97**	**Eventing** **N = 27**	**General Purpose** **N = 27**	**Racing** **N = 88**	**Show Jumping** **N = 47**	**Endurance** **N = 55**	
				**N and (%) of Limbs**	**N and (%) of Limbs**	**N and (%) of Limbs**	**N and (%) of Limbs**	**N and (%) of Limbs**	**N and (%) of Limbs**	***p* Value**
**MC/T 3**	**Cortex**	*Location*	Dorsal	14 (14.4)	8 (29.6)	5 (18.5)	**25 (28.4)**	6 (12.8)	4 (7.3)	**0.011**
			Palmar/Plantar	6 (6.2)	5 (18.5)	4 (14.8)	**28 (31.8)**	4 (8.5)	4 (7.3)	**<0.001**
			Medial	11 (11.3)	8 (29.6)	4 (14.8)	**29 (33)**	3 (6.4)	3 (5.5)	**<0.001**
			Lateral	2 (2.1)	4 (14.8)	3 (11.1)	**26 (29.6)**	1 (2.1)	3 (5.5)	**<0.001**
		*Aspect*	Periosteal	12 (12.4)	4 (14.8)	5 (18.5)	**29 (33)**	6 (12.8)	1 (1.8)	**<0.001**
			Endosteal	12 (12.4)	7 (25.9)	7 (25.9)	**35 (39.8)**	7 (14.9)	6 (10.9)	**<0.001**
			All	13 (13.4)	5 (18.5)	5 (18.5)	**29 (33)**	6 (12.8)	1 (1.8)	**<0.001**
		*Pathology* *type*	Sclerosis	10 (10.3)	6 (22.2)	5 (18.5)	**25 (28.4)**	4 (8.5)	5 (9.1)	**0.004**
			Fluid (hyperintensity)	9 (9.3)	3 (11.1)	2 (7.4)	**27 (30.7)**	4 (8.5)	5 (9.1)	**<0.001**
	**Trabecular** **bone**	*Location*	Palmar/Plantar	19 (19.6)	7 (25.9)	6 (22.2)	**60 (68.2)**	8 (17)	**40 (72.7)**	**<0.001**
			Medial	52 (53.6)	11 (40.7)	9 (33.3)	**57 (64.8)**	22 (46.8)	30 (54.6)	**0.042**
			Lateral	17 (17.5)	7 (25.9)	5 (18.5)	**53 (60.2)**	6 (12.8)	26 (47.3)	**<0.001**
		*Pathology* *type*	Sclerosis	45 (46.4)	10 (37)	10 (37)	54 (61.4)	18 (38.3)	**39 (70.9)**	**<0.001**
			Fluid (hyperintensity)	45 (46.4)	5 (18.5)	6 (22.2)	52 (59.1)	18 (38.3)	**42 (76.4)**	**<0.001**
			Fracture incomplete	-	-	1 (3.7)	2 (2.3)	-	**10 (18.2)**	**<0.001**
			Hypervascularisation	16 (16.5)	6 (22.2)	1 (3.7)	**59 (67.1)**	6 (12.8)	26 (47.3)	**<0.001**
**P1**	**Cortex**	*Location*	Dorsal	24 (24.7)	5 (18.5)	4 (14.8)	25 (28.4)	10 (21.3)	**24 (43.6)**	**0.037**
			Palmar/Plantar	1 (1)	1 (3.7)	**4 (14.8)**	5 (5.7)	3 (6.4)	-	**0.017**
			Lateral	2 (2.1)	-	**5 (18.5)**	7 (7.9)	2 (4.3)	4 (7.3)	**0.022**
		*Aspect*	Endosteal	27 (27.8)	5 (18.5)	7 (25.9)	28 (31.8)	10 (21.3)	**27 (49.1)**	**0.02**
		*Pathology* *type*	Irregularity	24 (24.7)	5 (18.5)	7 (25.9)	17 (19.3)	6 (12.8)	**26 (47.3)**	**0.001**
**PSB**	**Medial**	*Location*	Axial	12 (12.4)	4 (14.8)	2 (7.4)	**26 (29.6)**	6 (12.8)	6 (10.9)	**0.008**
			Abaxial	28 (28.9)	3 (11.1)	2 (7.4)	31 (35.2)	13 (27.7)	21 (38.2)	**0.014**
			Proximal	15 (15.5)	4 (14.8)	2 (7.4)	**26 (29.6)**	6 (12.8)	6 (10.9)	**0.008**
			Distal	16 (16.5)	1 (3.7)	3 (11.1)	**24 (27.3)**	7 (14.9)	5 (9.1)	**0.019**
			Diffuse	9 (9.3)	2 (7.4)	3 (11.1)	**30 (34.1)**	3 (6.4)	8 (14.6)	**<0.001**
		*Pathology* *type*	Irregularity	23 (23.7)	5 (18.5)	-	9 (10.2)	8 (17)	**16 (29.1)**	**0.006**
			Sclerosis	6 (6.2)	1 (3.7)	2 (7.4)	**23 (26.1)**	3 (6.4)	3 (5.5)	**<0.001**
			Fluid (hyperintensity)	17 (17.5)	1 (3.7)	2 (7.4)	24 (27.3)	7 (14.9)	**17 (30.9)**	**0.009**
	**Lateral**	*Location*	Abaxial	24 (24.7)	1 (3.7)	2 (7.4)	24 (27.3)	8 (17)	**18 (32.7)**	**0.013**
			Distal	9 (9.3)	-	3 (11.1)	**20 (22.7)**	7 (14.9)	5 (9.1)	**0.018**
			Diffuse	7 (7.2)	1 (3.7)	2 (7.4)	**25 (28.4)**	4 (8.5)	6 (10.9)	**<0.001**
		*Pathology* *type*	Irregularity	18 (18.6)	3 (11.1)	-	4 (4.6)	3 (6.4)	**14 (25.5)**	**<0.001**
			Sclerosis	7 (7.2)	1 (3.7)	1 (3.7)	**15 (17.1)**	-	4 (7.3)	**0.011**
**(b)** **Anatomical Location** **Joint**			**MRI Features**	**N and (%) of Limbs** **DRE (N = 97)**	**N and (%) of Limbs** **EVE (N = 27)**	**N and (%) of Limbs** **GP (N = 27)**	**N and (%) of Limbs** **RAC (N = 88)**	**N and (%) of Limbs** **SJ (N = 47)**	**N and (%) of Limbs** **END (N = 55)**	***p***
**MC/MTP**		*Location*	Dorsal P1	82 (84.5)	17 (63)	15 (55.6)	56 (63.6)	40 (85.1)	42 (76.4)	**0.001**
			Palmar/Plantar P1	16 (16.5)	5 (18.5)	7 (25.9)	11 (12.5)	**17 (36.2)**	4 (7.3)	**0.003**
			Palmar/Plantar MC/T 3	21 (21.7)	12 (44.4)	5 (18.5)	**63 (71.6)**	16 (34)	**43 (78.2)**	**<0.001**
			Medial P1	50 (51.6)	14 (51.9)	9 (33.3)	31 (35.2)	27 (57.5)	15 (27.3)	**0.006**
			Lateral MC/T3	21 (21.7)	10 (37)	7 (25.9)	**48 (54.6)**	17 (36.2)	25 (45.5)	**<0.001**
			Axial P1 (sagittal groove)	**58 (59.8)**	11 (40.7)	7 (25.9)	23 (26.1)	28 (59.6)	26 (47.3)	**<0.001**
			Medial Parasagittal groove	13 (13.4)	2 (7.4)	1 (3.7)	**32 (36.4)**	5 (10.6)	17 (30.9)	**<0.001**
			Lateral Parasagittal groove	7 (7.2)	-	1 (3.7)	**32 (36.4)**	4 (8.5)	**25 (45.5)**	**<0.001**
			Axial MC/T 3 (sagittal ridge)	64 (66)	14 (51.9)	4 (14.8)	36 (40.9)	25 (53.2)	38 (69.1)	**<0.001**
		*Pathology* *type*	Subchondral bone Irregularity	51 (52.6)	14 (51.9)	9 (33.3)	43 (48.9)	27 (57.5)	**43 (78.2)**	**0.002**
			Subchondral bone defect	19 (19.6)	3 (11.1)	1 (3.7)	**35 (39.8)**	7 (14.9)	8 (14.6)	**<0.001**
			Fissure	5 (5.2)	1 (3.7)	-	**38 (43.2)**	-	**22 (40)**	**<0.001**
			Osteophyte	**81 (83.5)**	11 (40.7)	14 (51.9)	18 (20.5)	35 (74.5)	**44 (80)**	**<0.001**
			POD	1 (1)	-	-	**15 (17.1)**	-	-	**<0.001**
			Distension	84 (86.6)	16 (59.3)	16 (59.3)	36 (40.9)	40 (85.1)	**53 (96.4)**	**<0.001**
			Chronic synovitis	75 (77.3)	18 (66.7)	16 (59.3)	28 (31.8)	33 (70.2)	**49 (89.1)**	**<0.001**
			Capsulitis	17 (17.5)	**11 (40.7)**	6 (22.2)	21 (23.9)	**17 (36.2)**	3 (5.5)	**<0.001**
**DFTS**		*Pathology* *Type*	Distension	12 (12.4)	3 (11.1)	**5 (18.5)**	2 (2.3)	3 (6.4)	1 (1.8)	**0.013**
			Chronic synovitis	**10 (10.3)**	3 (11.1)	3 (11.1)	1 (1.1)	2 (4.3)	1 (1.8)	**0.041**
**(c)** **Anatomical Location** **Soft Tissue**			**MRI Features**	**N and (%) of Limbs** **DRE (N = 97)**	**N and (%) of Limbs** **EVE (N = 27)**	**N and (%) of Limbs** **GP (N = 27)**	**N and (%) of Limbs** **RAC (N = 88)**	**N and (%) of Limbs** **SJ (N = 47)**	**N and (%) of Limbs** **END (N = 55)**	***p* value**
**Med SB**		*Location*	Insertion	19 (19.6)	4 (14.8)	3 (11.1)	5 (5.7)	**14 (29.8)**	**17 (30.9)**	**<0.001**
			Body	45 (46.4)	7 (25.9)	11 (40.7)	18 (20.5)	21 (44.7)	**34 (61.8)**	**<0.001**
		*Part of the* *Branch* *affected*	Margin/periphery	40 (41.2)	6 (22.2)	5 (18.5)	14 (15.9)	19 (40.4)	**29 (52.7)**	**<0.001**
			Central/core	33 (34)	5 (18.5)	3 (11.1)	13 (14.7)	16 (34)	21 (38.2)	**0.003**
		*Area of the* *Margin* *affected*	Axial	38 (39.2)	5 (18.5)	7 (25.9)	15 (17.1)	15 (31.9)	**28 (50.9)**	**<0.001**
		*Pathology* *type*	Defect	22 (22.7)	3 (11.1)	3 (11.1)	6 (6.8)	4 (8.5)	**29 (52.7)**	**<0.001**
			Fluid-based hyperintensity	40 (41.2)	10 (37)	9 (33.3)	13 (14.8)	19 (40.4)	**28 (50.9)**	**<0.001**
			Enlargement	21 (21.7)	4 (14.8)	7 (25.9)	12 (13.6)	**18 (38.3)**	11 (20)	**0.032**
**Lat SB**		*Location*	Insertion	**23 (23.7)**	4 (14.8)	2 (7.4)	3 (3.4)	8 (17)	10 (18.2)	**0.004**
			Body	32 (33)	11 (40.7)	6 (22.2)	10 (11.4)	16 (34)	16 (29.1)	**0.005**
		*Part of the* *branch* *affected*	Margin/periphery	**30 (30.9)**	5 (18.5)	4 (14.8)	8 (9.1)	10 (21.3)	16 (29.1)	**0.007**
			Central/core	26 (26.8)	8 (29.6)	3 (11.1)	9 (10.2)	14 (29.8)	10 (18.2)	**0.019**
			Entire branch	12 (12.4)	4 (14.8)	3 (11.1)	2 (2.3)	8 (17)	2 (3.6)	**0.024**
		*Area of the* *Margin* *affected*	Axial	**31 (32)**	6 (22.2)	5 (18.5)	7 (8)	10 (21.3)	15 (27.3)	**0.005**
		*Pathology* *type*	Fluid-based hyperintensity	33 (34)	11 (40.7)	7 (25.9)	10 (11.4)	18 (38.3)	13 (23.6)	**0.002**
			Enlargement	20 (20.1)	4 (14.8)	3 (11.1)	2 (2.3)	11 (23.4)	8 (14.6)	**0.004**
**LCL**		*Location*	Dorsal	9 (9.3)	1 (3.7)	**8 (29.6)**	4 (4.6)	3 (6.4)	1 (1.8)	**<0.001**
			Palmar/plantar	9 (9.3)	-	**7 (25.9)**	4 (4.6)	2 (4.3)	2 (1.8)	**<0.001**
			Axial	10 (10.3)	2 (7.4)	**8 (29.6)**	4 (4.6)	5 (10.6)	1 (1.8)	**<0.001**
			Abaxial	9 (9.3)	-	**7 (25.9)**	3 (3.4)	2 (4.3)	1 (1.8)	**<0.001**
			Entire ligament	8 (8.3)	-	**7 (25.9)**	2 (2.3)	2 (4.3)	1 (1.8)	**<0.001**
		*Level*	Superficial (long)	9 (9.3)	1 (3.7)	**8 (29.6)**	4 (4.6)	4 (8.5)	1 (1.8)	**<0.001**
			Deep (short)	9 (9.3)	2 (7.4)	**8 (29.6)**	4 (4.6)	5 (10.6)	1 (1.8)	**<0.001**
			Origin	7 (7.2)	1 (3.7)	**6 (22.2)**	4 (4.6)	3 (6.4)	1 (1.8)	**0.015**
			Body	5 (5.2)	1 (3.7)	**8 (29.6)**	2 (2.3)	3 (6.4)	1 (1.8)	**<0.001**
			Insertion	6 (6.2)	-	**4 (14.8)**	2 (2.3)	1 (2.1)	1 (1.8)	**0.034**
		*Pathology* *type*	Fluid-based hyperintensity	7 (7.2)	2 (7.4)	**9 (33.3)**	4 (4.6)	5 (10.6)	1 (1.8)	**<0.001**
			Enlargement	8 (8.3)	1 (3.7)	**8 (29.6)**	4 (4.6)	3 (6.4)	1 (1.8)	**<0.001**
**SDSL**		*Location*	Dorsal	8 (8.3)	-	-	-	-	1 (1.8)	**0.004**
			Palmar/plantar	**7 (7.2)**	-	-	-	-	1 (1.8)	**0.012**
			Medial	**9 (9.3)**	-	1 (3.7)	-	-	1 (1.8)	**0.004**
			Entire ligament	**6 (6.2)**	-	-	-	-	-	**0.009**
		*Level*	Body	**11 (11.3)**	1 (3.7)	-	-	-	1 (1.8)	**<0.001**
		*Pathology* *type*	Irregularity	**7 (7.2)**	-	-	-	-	1 (1.8)	**0.012**
			Fluid-based hyperintensity	**7 (7.2)**	-	-	-	-	-	**0.003**
**Med ODSL**		*Location*	Palmar/plantar	**15 (15.5)**	3 (11.1)	3 (11.1)	2 (2.3)	4 (8.5)	2 (3.6)	**0.028**
			Axial	**25 (25.8)**	1 (3.7)	3 (11.1)	2 (2.3)	**13 (27.7)**	5 (9.1)	**<0.001**
			Abaxial	**16 (16.5)**	2 (7.4)	1 (3.7)	2 (2.3)	2 (4.3)	2 (3.6)	**0.004**
			Entire ligament	**14 (14.4)**	**5 (18.5)**	1 (3.7)	3 (3.4)	2 (4.3)	-	**0.001**
		*Level*	Body	**27 (27.8)**	5 (18.5)	4 (14.8)	3 (3.4)	12 (25.5)	5 (9.1)	**<0.001**
		*Pathology* *type*	Irregularity	**16 (16.5)**	-	3 (11.1)	-	10 (21.3)	4 (7.3)	**<0.001**
			Enlargement	**17 (17.5)**	3 (11.1)	2 (7.4)	1 (1.1)	3 (6.4)	1 (1.8)	**<0.001**
			Fluid-based hyperintensity	**27 (27.8)**	5 (18.5)	3 (11.1)	2 (2.3)	7 (14.9)	6 (10.9)	**<0.001**
**Lat ODSL**		*Location*	Dorsal	**15 (15.5)**	3 (11.1)	5 (18.5)	-	3 (6.4)	1 (1.8)	**<0.001**
			Palmar/plantar	**17 (17.5)**	3 (11.1)	2 (7.4)	-	4 (8.5)	2 (3.6)	**<0.001**
			Axial	**21 (21.7)**	3 (11.1)	6 (22.2)	-	7 (14.9)	3 (5.5)	**<0.001**
			Abaxial	**16 (16.5)**	2 (7.4)	3 (11.1)	-	3 (6.4)	1 (1.8)	**<0.001**
			Entire ligament	**15 (15.5)**	2 (7.4)	4 (14.8)	-	2 (4.3)	-	**<0.001**
		*Level*	Bone ligament interface	2 (2.1)	-	**2 (7.4)**	-	-	-	**0.031**
			Body	**20 (20.6)**	3 (11.1)	6 (22.2)	-	8 (17)	4 (7.3)	**<0.001**
		*Pathology* *type*	Irregularity	**12 (12.4)**	-	5 (18.5)	-	4 (8.5)	3 (5.5)	**0.002**
			Enlargement	**14 (14.3)**	3 (11.1)	5 (18.5)	-	4 (8.5)	4 (7.3)	**0.007**
			Fluid-based hyperintensity	**20 (20.6)**	3 (11.1)	4 (14.8)	-	6 (12.8)	4 (7.3)	**<0.001**
**DDFT**		*Location*	Lateral	-	1 (3.7)	**2 (7.4)**	-	-	1 (1.8)	**0.019**
		*Level*	Proximal to the PSB	-	1 (3.7)	**2 (7.4)**	-	1 (2.1)	1 (1.8)	**0.019**
			Distal to the PSB	-	**2 (7.4)**	1 (3.7)	-	-	-	**0.003**
		*Pathology* *type*	Irregularity	-	**2 (7.4)**	**2 (7.4)**	-	1 (2.1)	1 (1.8)	**0.021**
			Enlargement	-	**1 (3.7)**	-	-	-	-	**0.04**
			Adhesions	-	**2 (7.4)**	1 (3.7)	-	-	1 (1.8)	**0.019**
			Chronic fibrosis	-	-	**3 (11.1)**	-	1 (2.1)	-	**<0.001**
**SDFT**		*Level*	Proximal to the PSB	1 (1)	-	**2 (7.4)**	-	2 (4.3)	-	**0.038**
		*Pathology* *type*	Enlargement	1 (1)	-	**2 (7.4)**	-	2 (4.3)	-	**0.038**

MCP j = metacarpophalangeal joint; MTP j = metatarsophalangeal joint; MC 3 = third metacarpal bone; MT 3 = third metatarsal bone; P1 = proximal phalanx; PSB= proximal sesamoid bones; POD = palmar/plantar osteochondral disease; DFTS = digital flexor tendon sheath; Med SB = medial suspensory branch; Lat SB = lateral suspensory branch; MCL = medial collateral ligament of the metacarpophalangeal/metatarsophalangeal joint; LCL = lateral collateral ligament of the metacarpophalangeal/metatarsophalangeal joint; SDSL = straight distal sesamoidean ligament; MODSL = medial oblique distal sesamoidean ligament; LODSL = lateral oblique distal sesamoidean ligament; DDFT = deep digital flexor tendon; SDFT = superficial digital flexor tendon.

## Data Availability

Anonymised data available on request to the first author.

## Supplementary Materials

The following supporting information can be downloaded at https://www.mdpi.com/article/10.3390/ani14131866/s1: Supplementary File S1: Features recorded included structure affected (bone, joint or soft tissue), location and type of MRI abnormality identified. Supplementary File S2: Tables displaying comparisons of the lesion distribution between the MCP and MTP regions. Supplementary File S3: Tables displaying comparisons of lesion distribution between horses performing different sports activities.
