# Supplementary material for: Lesion Distribution in the Metacarpophalangeal and Metatarsophalangeal Region of 341 Horses Using Standing Magnetic Resonance Imaging
Source: Animals (Basel). 2024 Jun 25;14(13):1866. doi: 10.3390/ani14131866 (PMC11240673; doi:10.3390/ani14131866)
Supplement: Supplementary file 1 [file animals-14-01866-s001.zip › animals-3012424-s1.pdf]

Supplementary file: MRI Features recorded

<u>Proximal phalanx</u>:

- Cortical bone:
  - Location: dorsal, palmar/plantar, axial (sagittal groove), medial and lateral
  - Aspect: periosteal, endosteal, all
  - Pathology type: irregular, sclerosis, hyperintensity on fluid-based sequencies, complete and incomplete fracture, microfracture, avulsion, osseous cyst like lesion
- Trabecular bone:
  - Location: dorsal, palmar/plantar, axial (sagittal groove), medial and lateral
  - Pathology type: irregular, sclerosis, hyperintensity on fluid-based sequencies, complete and incomplete fracture, microfracture, avulsion, osseous cyst like lesion

<u>Third metacarpal/metatarsal bone (MC/MT 3)</u>:

- Cortical bone:
  - Location: dorsal, palmar/plantar, axial (sagittal ridge), medial and lateral condyle
  - Aspect: periosteal, endosteal, all
  - Pathology type: irregular, sclerosis, hyperintensity on fluid-based sequencies, complete and incomplete fracture, microfracture, avulsion, osseous cyst like lesion
- Trabecular bone:
  - Location: dorsal, palmar/plantar, axial (sagittal ridge), medial and lateral condyle
  - Pathology type: irregular, sclerosis, hyperintensity on fluid-based sequencies, complete and incomplete fracture, microfracture, avulsion, osseous cyst like lesion

<u>Second and Fourth metacarpal/metatarsal bone (MC/MT 2/4):</u>

- Cortical bone:
  - Location: dorsal, palmar/plantar, medial and lateral
  - Aspect: Periosteal, endosteal, all
  - Pathology type: irregular, sclerosis, hyperintensity on fluid-based sequencies, complete and incomplete fracture, microfracture, syndesmopathy
- Trabecular bone:
  - Location: dorsal, palmar/plantar, medial and lateral
  - Pathology type: irregular, sclerosis, hyperintensity on fluid-based sequencies, complete and incomplete fracture, microfracture, syndesmopathy

<u>Medial and lateral proximal sesamoid bone (PSB):</u>

  - Location: axial, abaxial, proximal, distal, diffuse
  - Pathology type: irregular, sclerosis, fracture, avulsion, hyperintensity on fluid-based sequencies, osseous cyst like lesion, sesamoiditis, luxation

<u>Joint:</u>

<u>Metacarpophalangeal/metatarsophalangeal joint (MCP/MTP j):</u>

  - Location: dorsal proximal phalanx, dorsal third metacarpal/metatarsal bone, palmar/plantar proximal phalanx, palmar/plantar third metacarpal/metatarsal bone, medial glenoid proximal phalanx, medial condyle third metacarpal/metatarsal bone,

lateral glenoid proximal phalanx, lateral condyle third metacarpal/metatarsal bone, sagittal ridge, sagittal groove, lateral parasagittal groove, medial parasagittal groove, proximal phalanx, distal third metacarpal/metatarsal bone
- Pathology type: subchondral bone irregularity, subchondral bone sclerosis, hyperintensity on fluid-based sequencies, cartilage irregularity/fibrillation, fissure, osteophyte, palmar/plantar osteochondral disease, distension, capsulitis, chronic synovitis, invagination/osseous cyst like lesion, osteochondrosis, osteochondral fragment

Digital flexor tendon sheath (DFTS):

- Location: collateral recess, dorsoproximal recess, palmar/plantar distal recess
- Pathology type: distension, acute and chronic tenosynovitis

Soft tissues:

Medial and lateral suspensory branch (Med/Lat SB)

- Location: insertion, body
- Part of the branch affected: margin/periphery, central, entire branch
- Area of the margin affected: dorsal, palmar/plantar, axial, abaxial
- Pathology type: defect, hyperintensity on fluid-based sequencies, enlargement, split. adhesion, avulsion, chronic fibrosis

Medial and lateral collateral ligament of the metacarpophalangeal/metatarsophalangeal joint (MCP/MTP j):

- Location: dorsal, palmar/plantar, abaxial, axial, entire ligament
- Level: superficial (long), deep (short), origin, body, insertion, entire ligament
- Pathology type: defect, hyperintensity on fluid-based sequencies, enlargement, split. avulsion, chronic fibrosis, structural abnormality

Palmar/Plantar annular ligament (PAL):

- Location: dorsal, palmar/plantar, medial, lateral, entire ligament
- Level: proximal, distal
- Pathology type: defect, hyperintensity on fluid-based sequencies, enlargement, split. chronic fibrosis, structural abnormality

Common digital extensor tendon (CDET):

- Location: dorsal, palmar/plantar, medial, lateral, entire ligament
- Pathology type: defect, hyperintensity on fluid-based sequencies, enlargement, rupture. chronic fibrosis, structural abnormality

Deep digital flexor tendon (DDFT):

- Location: dorsal, palmar/plantar, medial, lateral, entire ligament
- Level: proximal to the proximal sesamoid bones, at the level of the proximal sesamoid bones, distal to the proximal sesamoid bones, body

- Pathology type: defect, hyperintensity on fluid-based sequencies, enlargement, rupture, chronic fibrosis, structural abnormality, dystrophic mineralization, core lesion, split, adhesions

Superficial digital flexor tendon (SDFT):

- Location: dorsal, palmar/plantar, medial, lateral, entire ligament
- Level: proximal to the proximal sesamoid bones, at the level of the proximal sesamoid bones, distal to the proximal sesamoid bones, body
- Pathology type: defect, hyperintensity on fluid-based sequencies, enlargement, rupture, chronic fibrosis, structural abnormality, dystrophic mineralization, core lesion, split, adhesions

Manica flexoria (MF):

- Location: dorsal, palmar/plantar, medial, lateral, entire manica
- Pathology type: defect, hyperintensity on fluid-based sequencies, enlargement, rupture, chronic fibrosis, structural abnormality, split, adhesions

Intersesamoidean ligament (ISL):

- Location: dorsal, palmar/plantar, medial, lateral, entire ligament
- Level: bone/ligament interface, body
- Pathology type: defect, hyperintensity on fluid-based sequencies, enlargement, chronic fibrosis, structural abnormality, split, adhesions

Medial and lateral oblique distal sesamoidean ligament (Med/Lat ODSL):

- Location: dorsal, palmar/plantar, abaxial, axial, entire ligament
- Level: bone/ligament interface, body
- Pathology type: defect, hyperintensity on fluid-based sequencies, enlargement, rupture, chronic fibrosis, structural abnormality, dystrophic mineralization, core lesion, split, adhesions

Medial and lateral extensor branch of the suspensory ligament:

- Location: dorsal, palmar/plantar, abaxial, axial, entire ligament
- Level: origin, body, insertion, entire ligament
- Pathology type: defect, hyperintensity on fluid-based sequencies, enlargement, rupture, avulsion

Medial and lateral collateral sesamoidean ligament:

- Location: dorsal, palmar/plantar, abaxial, axial, entire ligament
- Level: origin, body, insertion, entire ligament
- Pathology type: defect, hyperintensity on fluid-based sequencies, enlargement, rupture, avulsion
