# Supplementary material for: Lesion Distribution in the Metacarpophalangeal and Metatarsophalangeal Region of 341 Horses Using Standing Magnetic Resonance Imaging
Source: Animals (Basel). 2024 Jun 25;14(13):1866. doi: 10.3390/ani14131866 (PMC11240673; doi:10.3390/ani14131866)
Supplement: Supplementary file 1 [file animals-14-01866-s001.zip › Supplementary File 2-Tables comparison FL Vs HL.pdf]

## Supplementary File 2

MRI features from horses undergoing standing magnetic resonance imaging of the metacarpophalangeal/metatarsophalangeal region with number (N) and proportion (%) of forelimbs and hindlimbs affected. Features with significant differences in proportions between forelimbs and hindlimbs (Pearson's Chi-squared test) are shown, with the overrepresented category in bold, using a significance level of p<0.05. FL=forelimbs; HL=hindlimbs; MCP j=metacarpophalangeal joint; MTP j=metatarsophalangeal joint; MC 3=third metacarpal bone; MT 3=third metatarsal bone; P1=proximal phalanx; PSB=proximal sesamoid bone; POD=palmar/plantar osteochondral disease; DFTS=digital flexor tendon sheath; Med SB=medial suspensory branch; Lat SB=lateral suspensory branch; MCL=medial collateral ligament of the metacarpophalangeal/metatarsophalangeal joint; LCL=lateral collateral ligament of the metacarpophalangeal/metatarsophalangeal joint; SDSL=straight distal sesamoidean ligament; MODSL=medial oblique distal sesamoidean ligament; LODSL=lateral oblique distal sesamoidean ligament; DDFT=deep digital flexor tendon; SDFT=superficial digital flexor tendon.

**Table 2a**: Distribution and pathology type of **bone lesions** affecting the **forelimb and hindlimbs** of the metacarpophalangeal/metatarsophalangeal region with number (N) and proportion (%) of forelimbs and hindlimbs affected.
**Table 2b**: Distribution and pathology type of **metacarpophalangeal/metatarsophalangeal joint** and **digital flexor tendon sheath** lesions in forelimbs and hindlimbs.
**Table 2c**: Distribution and pathology type affecting the **soft tissues** of the metacarpophalangeal/metatarsophalangeal region with number (N) and proportion (%) of forelimbs and hindlimbs affected.

| **Table 2a Anatomical location Bone** | | | **MRI features** | **Number (N=259) and Proportion (%) of FL affected** | **Number (N=82) and Proportion (%) of HL affected** | **P value** |
|---|---|---|---|---|---|---|
| **MC/T 3** | **Cortex** | Location | Dorsal | 51 (19.7) | 11 (13.4) | |
| | | | Palmar/Plantar | 38 (14.7) | 13 (15.9) | |
| | | | Axial (sagittal ridge) | 30 (11.6) | 7 (8.5) | |
| | | | Medial | **51 (19.7)** | 7 (8.5) | **0.019** |
| | | | Lateral | 29 (11.2) | 10 (12.2) | |
| | | Aspect | Periosteal | 49 (18.9) | 9 (9.8) | |
| | | | Endosteal | 61 (23.6) | 13 (15.9) | |
| | | | All | 49 (18.9) | 10 (12.2) | |
| | | Pathology type | Irregularity | 24 (9.3) | 6 (7.3) | |
| | | | Sclerosis | 45 (17.4) | 10 (12.2) | |
| | | | Fluid (hyperintensity) | 42 (16.2) | 8 (9.8) | |
| | **Trabecular bone** | Location | Dorsal | 128 (49.5) | 31 (37.8) | |
| | | | Palmar/Plantar | 108 (41.7) | 32 (39) | |
| | | | Axial (sagittal ridge) | **68 (26.3)** | 12 (14.6) | **0.03** |

| | | | | | | |
|---|---|---|---|---|---|---|
| | | | | | | |
| | | | Medial | **151 (58.3)** | 30 (36.6) | **0.001** |
| | | | Lateral | 87 (33.6) | 27 (32.9) | |
| | | Pathology type | Irregularity | 34 (13.1) | 12 (14.6) | |
| | | | Sclerosis | 140 (54.1) | 36 (43.9) | |
| | | | Fluid (hyperintensity) | 134 (51.7) | 34 (41.5) | |
| | | | Fracture incomplete | 10 (3.9) | 3 (3.7) | |

| | | | | | | |
|---|---|---|---|---|---|---|
| | | | Hypervascularization | **97 (37.5)** | 17 (20.7) | **0.005** |
| **P1** | **Cortex** | Location | Dorsal | 65 (25.1) | 27 (32.9) | |
| | | | Palmar/Plantar | 8 (3.1) | 6 (7.3) | |
| | | | Axial (sagittal groove) | 41 (15.8) | 18 (22) | |
| | | | Medial | 30 (11.6) | **18 (22)** | **0.019** |
| | | | Lateral | 13 (5) | 7 (8.5) | |
| | | Aspect | Periosteal | 45 (17.4) | 18 (22) | |
| | | | Endosteal | 71 (27.4) | **33 (40.2)** | **0.028** |
| | | | All | 42 (16.2) | 18 (22) | |
| | | Pathology type | Irregularity | 56 (21.6) | **29 (35.4)** | **0.012** |
| | | | Sclerosis | 28 (10.8) | 15 (18.3) | |
| | | | Fluid (hyperintensity) | 39 (15.1) | 18 (22) | |
| | **Trabecular bone** | Location | Dorsal | 60 (23.2) | 24 (29.3) | |
| | | | Palmar/Plantar | 11 (4.2) | 7 (8.5) | |
| | | | Axial (sagittal groove) | 32 (12.4) | 12 (14.6) | |
| | | | Medial | 39 (15.1) | 15 (18.3) | |
| | | | Lateral | 15 (5.8) | 9 (11) | |
| | | Pathology type | Irregularity | 29 (11.2) | 16 (19.5) | |
| | | | Sclerosis | 32 (12.4) | 15 (18.3) | |
| | | | Fluid (hyperintensity) | 59 (22.8) | 20 (24.4) | |
| **PSB** | **Medial** | Location | Axial | **50 (19.3)** | 6 (7.3) | **0.011** |
| | | | Abaxial | **82 (31.7)** | 16 (19.5) | **0.034** |
| | | | Proximal | **56 (21.6)** | 7 (8.5) | **0.008** |
| | | | Distal | 47 (18.2) | 9 (11) | |
| | | | Diffuse | **50 (19.3)** | 5 (6.1) | **0.005** |
| | | Pathology type | Irregularity | 45 (17.4) | 16 (19.5) | |
| | | | Sclerosis | **36 (13.9)** | 2 (2.4) | **0.004** |
| | | | Fluid (hyperintensity) | 54 (20.9) | 14 (17.1) | |
| | **Lateral** | Location | Axial | 40 (15.4) | 8 (9.8) | |
| | | | Abaxial | 59 (22.8) | 18 (22) | |
| | | | Proximal | 38 (15.1) | 10 (12.2) | |
| | | | Distal | 35 (13.5) | 9 (11) | |
| | | | Diffuse | 38 (14.7) | 7 (8.5) | |
| | | Pathology type | Irregularity | 28 (10.8) | 14 (17.1) | |
| | | | Sclerosis | 18 (7) | 10 (12.2) | |
| | | | Fluid (hyperintensity) | 47 (18.2) | 20 (24.4) | |

| **Table 2b Anatomical location Joint** | | **MRI features** | **Number (N=259) and Proportion (%) of FL affected** | **Number (N=82) and Proportion (%) of HL affected** | **P value** |
|---|---|---|---|---|---|
| **MC/ MTP** | **Location** | Dorsal P1 | 193 (74.5) | 59 (72) | |
| | | Dorsal MC/T 3 | 190 (73.4) | 59 (72) | |
| | | Palmar/Plantar P1 | 42 (16.2) | 18 (22) | |

| | | | | | |
|---|---|---|---|---|---|
| | | Palmar/Plantar MC/T 3 | 124 (47.9) | 36 (43.9) | |
| | | Proximal P1 | 202 (78) | 60 (73.2) | |
| | | Distal MC/T 3 | 226 (87.3) | 71 (86.6) | |

| | | | | | |
|---|---|---|---|---|---|
| | | Medial P1 | **122 (47.1)** | 24 (29.3) | **0.004** |
| | | Medial MC/T 3 | **182 (70.3)** | 38 (46.3) | **<0.001** |
| | | Lateral P1 | 55 (21.2) | 17 (20.7) | |
| | | Lateral MC/T3 | 99 (38.2) | 29 (35.4) | |
| | | Axial P1 (sagittal groove) | 109 (42.1) | 44 (53.7) | |
| | | Medial parasagittal groove | 54 (20.9) | 16 (19.5) | |
| | | Lateral parasagittal groove | 50 (19.3) | 19 (23.2) | |
| | | Axial MC/T 3 (sagittal ridge) | 133 (51.4) | 48 (58.5) | |
| | **Pathology type** | Subchondral bone defect | 45 (28) | **17.4 (34.2)** | **<0.001** |
| | | Sclerosis | 196 (75.7) | 62 (75.6) | |
| | | Fluid (hyperintensity) | 204 (78.8) | 67 (81.8) | |
| | | Cartilage irregularity/fibrillation | 21 (8.1) | 5 (6.1) | |
| | | Fissure | 52 (20.1) | 14 (17.1) | |
| | | Osteophyte | 158 (61) | 45 (54.9) | |
| | | POD | 11 (4.3) | 5 (6.1) | |
| | | Distension | 176 (68) | **69 (84.2)** | **0.004** |
| | | Chronic synovitis | 158 (61) | **61 (74.4)** | **0.028** |
| | | Capsulitis | 53 (20.5) | 22 (26.8) | |
| **DFTS** | **Pathology type** | Distension | 14 (5.4) | **12 (14.6)** | **0.006** |
| | | Chronic synovitis | 10 (3.9) | **10 (12.2)** | **0.011** |

| **Table 2c Anatomical location Soft tissue** | | **MRI features** | **Number (N=259) and Proportion (%) of FL affected** | **Number (N=82) and Proportion (%) of HL affected** | **P value** |
|---|---|---|---|---|---|
| **Med SB** | Location | Insertion | 44 (17) | 18 (22) | |
| | | Body | 101 (39) | 35 (42.7) | |
| | Part of the branch affected | Margin/periphery | 82 (31.7) | 31 (37.8) | |
| | | Central/core | 64 (24.7) | 27 (32.9) | |
| | | Entire branch | 14 (5.4) | 9 (11) | |
| | Area of the margin affected | Dorsal | 18 (7) | 6 (7.3) | |
| | | Palmar/plantar | 32 (12.4) | 10 (12.2) | |
| | | Axial | 81 (31.3) | 27 (32.9) | |
| | | Abaxial | 17 (6.6) | **12 (14.6)** | **0.022** |
| | Pathology type | Defect | 52 (20.1) | 15 (18.3) | |
| | | Fluid-based hyperintensity | 89 (34.4) | 30 (36.6) | |
| | | Enlargement | 48 (18.5) | **25 (30.5)** | **0.021** |
| **Lat SB** | Location | Insertion | 33 (12.7) | 17 (20.7) | |
| | | Body | 69 (26.6) | 22 (26.8) | |
| | Part of the branch affected | Margin/periphery | 52 (20.1) | 21 (25.6) | |
| | | Central/core | 51 (19.7) | 19 (23.2) | |
| | | Entire branch | 20 (7.7) | 11 (13.4) | |
| | Area of the | Dorsal | 18 (7) | 10 (12.2) | |

| | | | | | |
|---|---|---|---|---|---|
| | margin affected | Palmar/plantar | 27 (10.4) | 12 (14.6) | |
| | | Axial | 53 (20.5) | 21 (25.6) | |
| | | Abaxial | 19 (7.3) | **12 (14.6)** | **0.045** |
| | Pathology type | Defect | 31 (12) | 11 (13.4) | |
| | | Fluid-based hyperintensity | 69 (26.6) | 23 (28.1) | |
| | | Enlargement | 30 (11.6) | **18 (22)** | **0.006** |
| **MCL** | Location | Dorsal | 5 (1.9) | 3 (3.7) | |
| | | Palmar/plantar | 4 (1.5) | 3 (3.7) | |
| | | Axial | 5 (1.9) | 3 (3.7) | |
| | | Abaxial | 5 (1.9) | 3 (3.7) | |
| | | Entire ligament | 4 (1.5) | 4 (4.9) | |
| | | Superficial (long) | 4 (1.5) | 4 (4.9) | |
| | | Deep (short) | 5 (1.9) | 4 (4.9) | |
| | | Origin | 3 (1.2) | 4 (4.9) | |
| | | Body | 3 (1.2) | 4 (4.9) | |
| | | Insertion | 2 (0.8) | **4 (4.9)** | **0.047** |
| | | Entire ligament | 1 (0.4) | 1 (1.2) | |
| | Pathology type | Fluid-based hyperintensity | 4 (1.5) | 4 (4.9) | |
| | | Enlargement | 3 (1.2) | 1 (1.2) | |
| **LCL** | Location | Dorsal | 14 (5.4) | **12 (14.6)** | **0.006** |
| | | Palmar/plantar | 11 (4.3) | **12 (14.6)** | **0.001** |
| | | Axial | 16 (6.2) | **14 (17.1)** | **0.002** |
| | | Abaxial | 10 (3.9) | **12 (14.6)** | **<0.001** |
| | | Entire ligament | 10 (3.9) | **10 (12.2)** | **0.011** |
| | Level | Superficial (long) | 14 (5.4) | **13 (15.9)** | **0.002** |
| | | Deep (short) | 15 (5.8) | **14 (17.1)** | **0.001** |
| | | Origin | 13 (5.2) | 9 (11) | |
| | | Body | 12 (4.6) | 6 (9.8) | |
| | | Insertion | 8 (3.1) | 6 (7.3) | |
| | | Entire ligament | 6 (2.3) | 4 (4.9) | |
| | Pathology type | Fluid-based hyperintensity | 15 (5.8) | **13 (15.9)** | **0.004** |
| | | Enlargement | 13 (5.2) | **12 (14.6)** | **0.004** |
| **SDSL** | Location | Dorsal | 5 (1.9) | 4 (4.9) | |
| | | Palmar/plantar | 4 (1.5) | 4 (4.9) | |
| | | Medial | 6 (2.3) | 5 (6.1) | |
| | | Lateral | 5 (1.9) | 2 (2.4) | |
| | | Entire ligament | 2 (0.8) | **4 (4.9)** | **0.047** |
| | Level | Bone ligament interface | 3 (1.2) | 2 (2.4) | |
| | | Body | 8 (3.1) | 5 (6.1) | |
| | Pathology type | Irregularity | 5 (1.9) | 3 (3.7) | |
| | | Enlargement | 4 (1.5) | 1 (1.2) | |
| | | Fluid-based hyperintensity | 4 (1.5) | 3 (3.7) | |

| | | | | | |
|---|---|---|---|---|---|
| | | Adhesions | 3 (1.2) | 2 (2.4) | |
| **Med ODSL** | Location | Dorsal | 16 (6.2) | 10 (12.2) | |
| | | Palmar/plantar | 19 (7.3) | 10 (12.2) | |
| | | Axial | 33 (12.7) | 16 (19.5) | |
| | | Abaxial | 16 (6.2) | 9 (11) | |
| | | Entire ligament | 17 (6.6) | 8 (9.8) | |
| | Level | Bone ligament interface | 4 (1.5) | 3 (3.7) | |
| | | Body | 42 (16.2) | 14 (17.1) | |
| | Pathology type | Irregularity | 21 (8.1) | 12 (14.6) | |
| | | Enlargement | 20 (7.7) | 7 (8.5) | |
| | | Fluid-based hyperintensity | 37 (14.3) | 13 (15.9) | |
| | | Adhesions | 2 (0.8) | 2 (2.4) | |
| **Lat ODSL** | Location | Dorsal | 17 (6.6) | 10 (12.2) | |
| | | Palmar/plantar | 17 (6.6) | **11 (13.4)** | **0.049** |
| | | Axial | 25 (9.7) | **15 (18.3)** | **0.034** |
| | | Abaxial | 17 (6.6) | 8 (9.8) | |
| | | Entire ligament | 14 (5.4) | 9 (11) | |
| | Level | Bone ligament interface | 3 (1.2) | 1 (1.2) | |
| | | Body | 30 (11.6) | 11 (13.4) | |
| | Pathology type | Irregularity | 15 (5.8) | 9 (11) | |
| | | Enlargement | 22 (8.5) | 8 (9.8) | |
| | | Fluid-based hyperintensity | 25 (9.7) | 12 (14.6) | |
| | | Adhesions | 2 (0.8) | 1 (1.2) | |
| **DDFT** | Location | Dorsal | 3 (1.2) | 1 (1.2) | |
| | | Palmar/plantar | 1 (0.4) | 1 (1.2) | |
| | | Medial | 1 (0.4) | 1 (1.2) | |
| | | Lateral | 4 (1.5) | | |
| | Level | Proximal to the PSB | 4 (1.5) | | |
| | | At the level of the PSB | 2 (0.8) | 1 (1.2) | |
| | | Distal to the PSB | 2 (0.8) | | |
| | | Body | 2 (0.8) | | |
| | Pathology type | Irregularity | 4 (1.5) | | |
| | | Enlargement | 1 (0.4) | | |
| | | Fluid-based hyperintensity | 2 (0.8) | | |
| | | Adhesions | 3 (1.2) | 1 (1.2) | |
| | | Chronic fibrosis | 3 (1.2) | | |
| **SDFT** | Location | Dorsal | 5 (1.9) | 3 (3.7) | |
| | | Palmar/plantar | 2 (0.8) | 1 (1.2) | |
| | | Medial | 6 (2.3) | 3 (3.7) | |
| | | Lateral | 3 (1.2) | | |
| | Level | Proximal to the | 3 (1.2) | 2 (2.4) | |

| | | | | | |
|---|---|---|---|---|---|
| | | PSB | | | |
| | | At the level of the PSB | 8 (3.1) | 4 (4.9) | |
| | | Distal to the PSB | 2 (0.8) | **5 (6.1)** | **0.012** |
| | | Body | 4 (1.5) | 1 (1.2) | |
| | Pathology type | Irregularity | 9 (3.5) | 5 (6.1) | |
| | | Enlargement | 3 (1.2) | 1 (1.2) | |
| | | Fluid-based hyperintensity | 8 (3.1) | 4 (4.9) | |
| | | Adhesions | 3 (1.2) | 4 (4.9) | |
| | | Chronic fibrosis | | 2 (2.4) | |
