# Supplementary material for: Lesion Distribution in the Metacarpophalangeal and Metatarsophalangeal Region of 341 Horses Using Standing Magnetic Resonance Imaging
Source: Animals (Basel). 2024 Jun 25;14(13):1866. doi: 10.3390/ani14131866 (PMC11240673; doi:10.3390/ani14131866)
Supplement: Supplementary file 1 [file animals-14-01866-s001.zip › Supplementary File 3-Tables comparison between sport disciplines.pdf]

Supplementary File 3

Comparison of lesion distribution between **horses doing different sports activities** are shown in **Table 3a**, **Table 3b** and **Table 3c**.

MRI features from horses undergoing standing magnetic resonance imaging of the metacarpophalangeal/metatarsophalangeal region from horses with a history of undertaking different sports with number (N) and proportion (%) of forelimbs and hindlimbs affected. Features with significant differences in proportions between different sports (Pearson's Chi-squared test) are shown with overrepresented category in bold and underrepresented underlined, using a significant level of $p<0.05$.
MCP j=metacarpophalangeal joint; MTP j=metatarsophalangeal joint; MC 3=third metacarpal bone; MT 3=third metatarsal bone; P1=proximal phalanx; PSB= proximal sesamoid bones; POD=palmar/plantar osteochondral disease; DFTS=digital flexor tendon sheath; Med SB=medial suspensory branch; Lat SB=lateral suspensory branch; MCL=medial collateral ligament of the metacarpophalangeal/metatarsophalangeal joint; LCL=lateral collateral ligament of the metacarpophalangeal/metatarsophalangeal joint; SDSL=straight distal sesamoidean ligament; MODSL=medial oblique distal sesamoidean ligament; LODSL=lateral oblique distal sesamoidean ligament; DDFT=deep digital flexor tendon; SDFT=superficial digital flexor tendon.

Table 3a: Distribution and pathology type of **bone lesions** of the metacarpophalangeal/metatarsophalangeal region from horses with a history of undertaking different sports with number (N) and proportion (%) of forelimbs and hindlimbs affected.
Table 3b: Distribution and pathology type of **metacarpophalangeal/metatarsophalangeal joint** and **digital flexor tendon sheath** lesions from horses with a history of undertaking different sports with number (N) and proportion (%) of forelimbs and hindlimbs affected.
Table 3c: Distribution and pathology type affecting the **soft tissues** of the metacarpophalangeal/metatarsophalangeal region from horses with a history of undertaking different sports with number (N) and proportion (%) of forelimbs and hindlimbs affected.

**Table 3a Anatomical location Bone**			**MRI features**	**Dressage N=97**	**Eventing N=27**	**General purpose N=27**	**Racing N=88**	**Show jumping N=47**	**Endurance N=55**	
				**N and (%) of limbs**	**N and (%) of limbs**	**N and (%) of limbs**	**N and (%) of limbs**	**N and (%) of limbs**	**N and (%) of limbs**	**P value**
**MC/T 3**	**Cortex**	*Location*	Dorsal	14 (14.4)	8 (29.6)	5 (18.5)	**25 (28.4)**	6 (12.8)	4 (7.3)	**0.011**
			Palmar/Plantar	6 (6.2)	5 (18.5)	4 (14.8)	**28 (31.8)**	4 (8.5)	4 (7.3)	**<.001**
			Axial (sagittal ridge)	7 (7.2)	5 (18.5)	3 (11.1)	15 (17)	5 (10.6)	2 (3.6)	
			Medial	11 (11.3)	8 (29.6)	4 (14.8)	**29 (33)**	3 (6.4)	3 (5.5)	**<.001**
			Lateral	2 (2.1)	4 (14.8)	3 (11.1)	**26 (29.6)**	1 (2.1)	3 (5.5)	**<.001**
		Aspect	Periosteal	12 (12.4)	4 (14.8)	5 (18.5)	**29 (33)**	6 (12.8)	1 (1.8)	**<.001**
			Endosteal	12 (12.4)	7 (25.9)	7 (25.9)	**35 (39.8)**	7 (14.9)	6 (10.9)	**<.001**
			All	13 (13.4)	5 (18.5)	5 (18.5)	**29 (33)**	6 (12.8)	1 (1.8)	**<.001**
		Pathology type	Irregularity	4 (4.1)	2 (7.4)	2 (7.4)	13 (14.8)	4 (8.5)	5 (9.1)	
			Sclerosis	10 (10.3)	6 (22.2)	5 (18.5)	**25 (28.4)**	4 (8.5)	5 (9.1)	**0.004**

			Fluid (hyperintensity)	9 (9.3)	3 (11.1)	2 (7.4)	**27 (30.7)**	4 (8.5)	5 (9.1)	**<.001**
	**Trabecular bone**	*Location*	Dorsal	49 (50.5)	10 (37)	10 (37)	44 (50)	21 (44.7)	25 (45.5)	
			Palmar/Plantar	19 (19.6)	7 (25.9)	6 (22.2)	**60 (68.2)**	8 (17)	**40 (72.7)**	**<.001**
			Axial (sagittal ridge)	24 (24.7)	4 (14.8)	3 (11.1)	25 (28.4)	10 (21.3)	14 (25.5)	
			Medial	52 (53.6)	11 (40.7)	9 (33.3)	**57 (64.8)**	22 (46.8)	30 (54.6)	**0.042**
			Lateral	17 (17.5)	7 (25.9)	5 (18.5)	**53 (60.2)**	6 (12.8)	26 (47.3)	**<.001**
		Pathology type	Irregularity	13 (13.4)	2 (7.4)	2 (7.4)	10 (11.4)	5 (10.6)	14 (25.5)	
			Sclerosis	45 (46.4)	10 (37)	10 (37)	54 (61.4)	18 (38.3)	**39 (70.9)**	**<.001**
			Fluid (hyperintensity)	45 (46.4)	5 (18.5)	6 (22.2)	52 (59.1)	18 (38.3)	42 (76.4)	**<.001**
			Fracture incomplete	-	-	1 (3.7)	2 (2.3)	-	**10 (18.2)**	**<.001**
			Hypervascularization	16 (16.5)	6 (22.2)	1 (3.7)	**59 (67.1)**	6 (12.8)	26 (47.3)	**<.001**
**P1**	**Cortex**	*Location*	Dorsal	24 (24.7)	5 (18.5)	4 (14.8)	25 (28.4)	10 (21.3)	**24 (43.6)**	**0.037**
			Palmar/Plantar	1 (1)	1 (3.7)	**4 (14.8)**	5 (5.7)	3 (6.4)	-	**0.017**
			Axial (sagittal groove)	15 (15.5)	3 (11.1)	2 (7.4)	15 (17.1)	7 (14.9)	17 (30.9)	
			Medial	15 (15.5)	3 (11.1)	4 (14.8)	15 (17.1)	7 (14.9)	4 (7.3)	
			Lateral	2 (2.1)	-	**5 (18.5)**	7 (7.9)	2 (4.3)	4 (7.3)	**0.022**
		Aspect	Periosteal	19 (19.6)	4 (14.8)	7 (25.9)	22 (25)	6 (12.8)	5 (9.1)	
			Endosteal	27 (27.8)	5 (18.5)	7 (25.9)	28 (31.8)	10 (21.3)	**27 (49.1)**	**0.02**
			All	19 (19.6)	4 (14.8)	6 (22.2)	21 (23.9)	6 (12.8)	4 (7.3)	
		Pathology type	Irregularity	24 (24.7)	5 (18.5)	7 (25.9)	17 (19.3)	6 (12.8)	**26 (47.3)**	**0.001**
			Sclerosis	12 (12.4)	4 (14.8)	4 (14.8)	15 (17.1)	4 (8.5)	4 (7.3)	
			Fluid (hyperintensity)	15 (15.5)	2 (7.4)	5 (18.5)	19 (21.6)	5 (10.6)	11 (20)	
	**Trabecular bone**	*Location*	Dorsal	27 (27.8)	3 (11.1)	3 (11.1)	26 (29.6)	12 (25.5)	13 (23.6)	
			Palmar/Plantar	4 (4.1)	1 (3.7)	4 (14.8)	4 (4.6)	3 (6.4)	2 (3.6)	
			Axial (sagittal groove)	10 (10.3)	1 (3.7)	2 (7.4)	12 (13.6)	8 (17)	11 (20)	
			Medial	21 (21.7)	3 (11.1)	2 (7.4)	14 (15.9)	10 (21.3)	4 (7.3)	
			Lateral	4 (4.1)	-	4 (14.8)	8 (9.1)	4 (8.5)	4 (7.3)	
		Pathology type	Irregularity	16 (16.5)	7 (25.9)	2 (7.4)	5 (5.7)	11 (23.5)	4 (7.3)	
			Sclerosis	14 (14.4)	5 (18.5)	3 (11.1)	4 (4.6)	16 (34.1)	5 (9.1)	
			Fluid (hyperintensity)	27 (27.8)	11 (40.7)	2 (7.4)	6 (6.8)	23 (49.1)	10 (18.2)	
**PSB**	**Medial**	*Location*	Axial	12 (12.4)	4 (14.8)	2 (7.4)	**26 (29.6)**	6 (12.8)	6 (10.9)	**0.008**
			Abaxial	28 (28.9)	3 (11.1)	2 (7.4)	31 (35.2)	13 (27.7)	21 (38.2)	**0.014**
			Proximal	15 (15.5)	4 (14.8)	2 (7.4)	**26 (29.6)**	6 (12.8)	6 (10.9)	**0.008**
			Distal	16 (16.5)	1 (3.7)	3 (11.1)	**24 (27.3)**	7 (14.9)	5 (9.1)	**0.019**
			Diffuse	9 (9.3)	2 (7.4)	3 (11.1)	**30 (34.1)**	3 (6.4)	8 (14.6)	**<.001**
		Pathology type	Irregularity	23 (23.7)	5 (18.5)	-	9 (10.2)	8 (17)	**16 (29.1)**	**0.006**
			Sclerosis	6 (6.2)	1 (3.7)	2 (7.4)	**23 (26.1)**	3 (6.4)	3 (5.5)	**<.001**

			Fluid (hyperintensity)	17 (17.5)	1 (3.7)	2 (7.4)	24 (27.3)	7 (14.9)	**17 (30.9)**	**0.009**
	**Lateral**	*Location*	Axial	9 (9.3)	3 (11.1)	2 (7.4)	20 (22.7)	7 (14.9)	7 (12.7)	
			Abaxial	24 (24.7)	1 (3.7)	2 (7.4)	24 (27.3)	8 (17)	**18 (32.7)**	**0.013**
			Proximal	15 (15.5)	1 (3.7)	2 (7.4)	20 (22.7)	6 (12.8)	5 (9.1)	
			Distal	9 (9.3)	-	3 (11.1)	**20 (22.7)**	7 (14.9)	5 (9.1)	**0.018**
			Diffuse	7 (7.2)	1 (3.7)	2 (7.4)	**25 (28.4)**	4 (8.5)	6 (10.9)	**<.001**
		Pathology type	Irregularity	18 (18.6)	3 (11.1)	-	4 (4.6)	3 (6.4)	**14 (25.5)**	**<.001**
			Sclerosis	7 (7.2)	1 (3.7)	1 (3.7)	**15 (17.1)**	-	4 (7.3)	**0.011**
			Fluid (hyperintensity)	19 (19.6)	-	2 (7.4)	23 (26.1)	7 (14.9)	16 (29.1)	

**Table 3b**<br>**Anatomical location**<br>**Joint**		**MRI features**	**N and (%) of limbs DRE (N=97)**	**N and (%) of limbs EVE (N=27)**	**N and (%) of limbs GP (N=27)**	**N and (%) of limbs RAC (N=88)**	**N and (%) of limbs SJ (N=47)**	**N and (%) of limbs END (N=55)**	**P value**
**MC/MTP**	*Location*	Dorsal P1	82 (84.5)	17 (63)	15 (55.6)	56 (63.6)	40 (85.1)	42 (76.4)	**0.001**
		Dorsal MC/T 3	85 (87.6)	19 (70.4)	14 (51.9)	54 (61.5)	35 (74.5)	42 (76.4)	
		Palmar/Plantar P1	16 (16.5)	5 (18.5)	7 (25.9)	11 (12.5)	**17 (36.2)**	4 (7.3)	**0.003**
		Palmar/Plantar MC/T 3	21 (21.7)	12 (44.4)	5 (18.5)	**63 (71.6)**	16 (34)	**43 (78.2)**	**<.001**
		Proximal P1	87 (89.7)	19 (70.4)	18 (66.7)	55 (62.5)	38 (80.9)	45 (81.8)	
		Distal MC/T 3	87 (89.7)	22 (81.5)	15 (59.3)	79 (89.8)	41 (87.2)	52 (94.6)	
		Medial P1	50 (51.6)	14 (51.9)	9 (33.3)	31 (35.2)	27 (57.5)	15 (27.3)	**0.006**
		Medial MC/T 3	67 (69.1)	14 (51.9)	12 (44.4)	61 (69.3)	33 (70.2)	33 (60)	
		Lateral P1	23 (23.7)	3 (11.1)	5 (18.5)	14 (15.9)	15 (31.9)	12 (21.8)	
		Lateral MC/T3	21 (21.7)	10 (37)	7 (25.9)	**48 (54.6)**	17 (36.2)	25 (45.5)	**<.001**
		Axial P1 (sagittal groove)	58 (59.8)	11 (40.7)	7 (25.9)	23 (26.1)	28 (59.6)	26 (47.3)	**<.001**
		Medial Parasagittal groove	13 (13.4)	2 (7.4)	1 (3.7)	**32 (36.4)**	5 (10.6)	17 (30.9)	**<.001**
		Lateral Parasagittal groove	7 (7.2)	-	1 (3.7)	**32 (36.4)**	4 (8.5)	**25 (45.5)**	**<.001**
		Axial MC/T 3 (sagittal ridge)	64 (66)	14 (51.9)	4 (14.8)	36 (40.9)	25 (53.2)	38 (69.1)	**<.001**
	Pathology type	Subchondral bone Irregularity	51 (52.6)	14 (51.9)	9 (33.3)	43 (48.9)	27 (57.5)	**43 (78.2)**	**0.002**
		Subchondral bone defect	19 (19.6)	3 (11.1)	1 (3.7)	**35 (39.8)**	7 (14.9)	8 (14.6)	**<.001**
		Sclerosis	74 (76.3)	19 (70.4)	14 (51.9)	66 (75)	35 (74.5)	50 (90.9)	
		Fluid (hyperintensity)	82 (84.5)	14 (51.9)	13 (48.2)	77 (87.5)	34 (72.3)	51 (92.7)	

		Cartilage irregularity/fibrillation	7 (7.2)	2 (7.4)	1 (3.7)	4 (4.6)	8 (17)	4 (7.3)	
		Fissure	5 (5.2)	1 (3.7)	-	**38 (43.2)**	-	**22 (40)**	**<.001**
		Osteophyte	**81 (83.5)**	11 (40.7)	14 (51.9)	18 (20.5)	35 (74.5)	**44 (80)**	**<.001**
		POD	1 (1)	-	-	**15 (17.1)**	-	-	**<.001**
		Distension	84 (86.6)	16 (59.3)	16 (59.3)	36 (40.9)	40 (85.1)	**53 (96.4)**	**<.001**
		Chronic synovitis	75 (77.3)	18 (66.7)	16 (59.3)	28 (31.8)	33 (70.2)	**49 (89.1)**	**<.001**
		Capsulitis	17 (17.5)	**11 (40.7)**	6 (22.2)	21 (23.9)	17 (36.2)	3 (5.5)	**<.001**
**DFTS**	*Pathology Type*	Distension	12 (12.4)	3 (11.1)	**5 (18.5)**	2 (2.3)	3 (6.4)	1 (1.8)	**0.013**
		Chronic synovitis	**10 (10.3)**	3 (11.1)	3 (11.1)	1 (1.1)	2 (4.3)	1 (1.8)	**0.041**

**Table 3c Anatomical location Soft tissue**		**MRI features**	**N and (%) of limbs DRE (N=97)**	**N and (%) of limbs EVE (N=27)**	**N and (%) of limbs GP (N=27)**	**N and (%) of limbs RAC (N=88)**	**N and (%) of limbs SJ (N=47)**	**N and (%) of limbs END (N=55)**	**P value**
**Med SB**	*Location*	Insertion	19 (19.6)	4 (14.8)	3 (11.1)	5 (5.7)	**14 (29.8)**	**17 (30.9)**	**<.001**
		Body	45 (46.4)	7 (25.9)	11 (40.7)	18 (20.5)	21 (44.7)	**34 (61.8)**	**<.001**
	Part of the Branch affected	Margin/periphery	40 (41.2)	6 (22.2)	5 (18.5)	14 (15.9)	19 (40.4)	**29 (52.7)**	**<.001**
		Central/core	33 (34)	5 (18.5)	3 (11.1)	13 (14.7)	16 (34)	21 (38.2)	**0.003**
		Entire branch	6 (6.2)	3 (11.1)	4 (14.8)	6 (6.8)	4 (8.5)	-	
	Area of the Margin affected	Dorsal	11 (11.3)	-	3 (11.1)	5 (5.7)	4 (8.5)	1 (1.8)	
		Palmar/plantar	15 (15.5)	4 (14.8)	5 (18.5)	7 (8)	8 (17)	3 (5.5)	
		Axial	38 (39.2)	5 (18.5)	7 (25.9)	15 (17.1)	15 (31.9)	**28 (50.9)**	**<.001**
		Abaxial	13 (13.4)	1 (3.7)	4 (14.8)	6 (6.8)	3 (6.4)	2 (3.6)	
	Pathology type	Defect	22 (22.7)	3 (11.1)	3 (11.1)	6 (6.8)	4 (8.5)	**29 (52.7)**	**<.001**
		Fluid-based hyperintensity	40 (41.2)	10 (37)	9 (33.3)	13 (14.8)	19 (40.4)	**28 (50.9)**	**<.001**
		Enlargement	21 (21.7)	4 (14.8)	7 (25.9)	12 (13.6)	**18 (38.3)**	11 (20)	**0.032**
**Lat SB**	*Location*	Insertion	**23 (23.7)**	4 (14.8)	2 (7.4)	3 (3.4)	8 (17)	10 (18.2)	**0.004**
		Body	32 (33)	11 (40.7)	6 (22.2)	10 (11.4)	16 (34)	16 (29.1)	**0.005**
	Part of the branch affected	Margin/periphery	**30 (30.9)**	5 (18.5)	4 (14.8)	8 (9.1)	10 (21.3)	16 (29.1)	**0.007**
		Central/core	26 (26.8)	8 (29.6)	3 (11.1)	9 (10.2)	14 (29.8)	10 (18.2)	**0.019**
		Entire branch	12 (12.4)	4 (14.8)	3 (11.1)	2 (2.3)	8 (17)	2 (3.6)	**0.024**
	Area of the Margin affected	Dorsal	13 (13.4)	3 (11.1)	3 (11.1)	1 (1.1)	4 (8.5)	4 (7.3)	
		Palmar/plantar	15 (15.5)	5 (18.5)	2 (7.4)	4 (4.6)	7 (14.9)	6 (10.9)	
		Axial	**31 (32)**	6 (22.2)	5 (18.5)	7 (8)	10 (21.3)	15 (27.3)	**0.005**
		Abaxial	14 (14.4)	4 (14.8)	2 (7.4)	2 (2.3)	5 (10.6)	4 (7.3)	
	Pathology type	Defect	18 (18.6)	2 (7.4)	-	6 (6.8)	1 (2.1)	15 (27.3)	
		Fluid-based hyperintensity	33 (34)	11 (40.7)	7 (25.9)	10 (11.4)	18 (38.3)	13 (23.6)	**0.002**
		Enlargement	20 (20.1)	4 (14.8)	3 (11.1)	2 (2.3)	11 (23.4)	8 (14.6)	**0.004**
**MCL**	*Location*	Dorsal	4 (4.1)	-	1 (3.7)	1 (1.1)	2 (4.3)	-	

		Palmar/plantar	4 (4.1)	-	1 (3.7)	1 (1.1)	1 (2.1)	-	
		Axial	5 (5.2)	-	1 (3.7)	1 (1.1)	1 (2.1)	-	
		Abaxial	4 (4.1)	-	1 (3.7)	1 (1.1)	2 (4.3)	-	
		Entire ligament	4 (4.1)	1 (3.7)	1 (3.7)	1 (1.1)	1 (2.1)	-	
		Superficial (long)	4 (4.1)	1 (3.7)	1 (3.7)	-	2 (4.3)	-	
		Deep (short)	5 (5.2)	1 (3.7)	1 (3.7)	1 (1.1)	1 (2.1)	-	
		Origin	4 (4.1)	1 (3.7)	-	1 (1.1)	1 (2.1)	-	
		Body	5 (5.2)	1 (3.7)	1 (3.7)	-	1 (2.1)	-	
		Insertion	5 (5.2)	1 (3.7)	-	-	-	-	
		Entire ligament	2 (2.1)	-	-	-	-	-	
	Pathology type	Fluid-based hyperintensity	5 (5.2)	1 (3.7)	-	-	2 (4.3)	-	
		Enlargement	1 (1)	1 (3.7)	1 (3.7)	-	1 (2.1)	-	
**LCL**	*Location*	Dorsal	9 (9.3)	1 (3.7)	**8 (29.6)**	4 (4.6)	3 (6.4)	1 (1.8)	**<.001**
		Palmar/plantar	9 (9.3)	-	**7 (25.9)**	4 (4.6)	2 (4.3)	2 (1.8)	**<.001**
		Axial	10 (10.3)	2 (7.4)	**8 (29.6)**	4 (4.6)	5 (10.6)	1 (1.8)	**<.001**
		Abaxial	9 (9.3)	-	**7 (25.9)**	3 (3.4)	2 (4.3)	1 (1.8)	**<.001**
		Entire ligament	8 (8.3)	-	**7 (25.9)**	2 (2.3)	2 (4.3)	1 (1.8)	**<.001**
	Level	Superficial (long)	9 (9.3)	1 (3.7)	**8 (29.6)**	4 (4.6)	4 (8.5)	1 (1.8)	**<.001**
		Deep (short)	9 (9.3)	2 (7.4)	**8 (29.6)**	4 (4.6)	5 (10.6)	1 (1.8)	**<.001**
		Origin	7 (7.2)	1 (3.7)	**6 (22.2)**	4 (4.6)	3 (6.4)	1 (1.8)	**0.015**
		Body	5 (5.2)	1 (3.7)	**8 (29.6)**	2 (2.3)	3 (6.4)	1 (1.8)	**<.001**
		Insertion	6 (6.2)	-	**4 (14.8)**	2 (2.3)	1 (2.1)	1 (1.8)	**0.034**
		Entire ligament	4 (4.1)	-	3 (11.1)	1 (1.1)	1 (2.1)	1 (1.8)	
	Pathology type	Fluid-based hyperintensity	7 (7.2)	2 (7.4)	**9 (33.3)**	4 (4.6)	5 (10.6)	1 (1.8)	**<.001**
		Enlargement	8 (8.3)	1 (3.7)	**8 (29.6)**	4 (4.6)	3 (6.4)	1 (1.8)	**<.001**
**SDSL**	*Location*	Dorsal	8 (8.3)	-	-	-	-	1 (1.8)	**0.004**
		Palmar/plantar	**7 (7.2)**	-	-	-	-	1 (1.8)	**0.012**
		Medial	**9 (9.3)**	-	1 (3.7)	-	-	1 (1.8)	**0.004**
		Lateral	5 (5.2)	1 (3.7)	-	-	-	1 (1.8)	
		Entire ligament	**6 (6.2)**	-	-	-	-	-	**0.009**
	Level	Bone ligament interface	3 (3.1)	-	-	-	-	-	
		Body	**11 (11.3)**	1 (3.7)	-	-	-	1 (1.8)	**<.001**
	Pathology type	Irregularity	**7 (7.2)**	-	-	-	-	1 (1.8)	**0.012**
		Enlargement	**4 (4.1)**	-	-	-	-	1 (1.8)	
		Fluid-based hyperintensity	**7 (7.2)**	-	-	-	-	-	**0.003**
		Adhesions	3 (3.1)	1 (3.7)	1 (3.7)	-	-	-	
**Med ODSL**	*Location*	Dorsal	14 (14.4)	2 (7.4)	1 (3.7)	2 (2.3)	4 (8.5)	3 (5.5)	
		Palmar/plantar	**15 (15.5)**	3 (11.1)	3 (11.1)	2 (2.3)	4 (8.5)	2 (3.6)	**0.028**
		Axial	**25 (25.8)**	1 (3.7)	3 (11.1)	2 (2.3)	**13 (27.7)**	5 (9.1)	**<.001**
		Abaxial	**16 (16.5)**	2 (7.4)	1 (3.7)	2 (2.3)	2 (4.3)	2 (3.6)	**0.004**
		Entire ligament	**14 (14.4)**	**5 (18.5)**	1 (3.7)	3 (3.4)	2 (4.3)	-	**0.001**

	Level	Bone ligament interface	4 (4.1)	1 (3.7)	1 (3.7)	1 (1.1)	-	-	
		Body	**27 (27.8)**	5 (18.5)	4 (14.8)	3 (3.4)	12 (25.5)	5 (9.1)	**<.001**
	Pathology type	Irregularity	**16 (16.5)**	-	3 (11.1)	-	10 (21.3)	4 (7.3)	**<.001**
		Enlargement	**17 (17.5)**	3 (11.1)	2 (7.4)	1 (1.1)	3 (6.4)	1 (1.8)	**<.001**
		Fluid-based hyperintensity	**27 (27.8)**	5 (18.5)	3 (11.1)	2 (2.3)	7 (14.9)	6 (10.9)	**<.001**
		Adhesions	3 (3.1)	-	1 (3.7)	-	-	-	
**Lat ODSL**	*Location*	Dorsal	**15 (15.5)**	3 (11.1)	5 (18.5)	-	3 (6.4)	1 (1.8)	**<.001**
		Palmar/plantar	**17 (17.5)**	3 (11.1)	2 (7.4)	-	4 (8.5)	2 (3.6)	**<.001**
		Axial	**21 (21.7)**	3 (11.1)	6 (22.2)	-	7 (14.9)	3 (5.5)	**<.001**
		Abaxial	**16 (16.5)**	2 (7.4)	3 ()11.1	-	3 (6.4)	1 (1.8)	**<.001**
		Entire ligament	**15 (15.5)**	2 (7.4)	4 (14.8)	-	2 (4.3)	-	**<.001**
	Level	Bone ligament interface	2 (2.1)	-	**2 (7.4)**	-	-	-	**0.031**
		Body	**20 (20.6)**	3 (11.1)	6 (22.2)	-	8 (17)	4 (7.3)	**<.001**
	Pathology type	Irregularity	**12 (12.4)**	-	5 (18.5)	-	4 (8.5)	3 (5.5)	**0.002**
		Enlargement	**14 (14.3)**	3 (11.1)	5 (18.5)	-	4 (8.5)	4 (7.3)	**0.007**
		Fluid-based hyperintensity	**20 (20.6)**	3 (11.1)	4 (14.8)	-	6 (12.8)	4 (7.3)	**<.001**
		Adhesions	-	-	2 (7.4)	-	1 (2.1)	-	
**DDFT**	*Location*	Dorsal	-	1 (3.7)	-	-	2 (4.3)	1 (1.8)	
		Palmar/plantar	-	1 (3.7)	1 (3.7)	-	-	-	
		Medial	-	-	-	-	1 (2.1)	-	
		Lateral	-	1 (3.7)	**2 (7.4)**	-	-	1 (1.8)	**0.019**
	Level	Proximal to the PSB	-	1 (3.7)	**2 (7.4)**	-	1 (2.1)	1 (1.8)	**0.019**
		At the level of the PSB	-	1 (3.7)	1 (3.7)	-	1 (2.1)	-	
		Distal to the PSB	-	**2 (7.4)**	1 (3.7)	-	-	-	**0.003**
		Body	-	-	1 (3.7)	-	2 (4.3)	-	
	Pathology type	Irregularity	-	**2 (7.4)**	**2 (7.4)**	-	1 (2.1)	1 (1.8)	**0.021**
		Enlargement	-	**1 (3.7)**	-	-	-	-	**0.04**
		Fluid-based hyperintensity	-	1 (3.7)	-	-	-	1 (1.8)	
		Adhesions	-	**2 (7.4)**	1 (3.7)	-	-	1 (1.8)	**0.019**
		Chronic fibrosis	-	-	**3 (11.1)**	-	1 (2.1)	-	**<.001**
**SDFT**	*Location*	Dorsal	2 (2.1)	-	1 (3.7)	2 (2.3)	1 (2.1)	2 (3.6)	
		Palmar/plantar	-	1 (3.7)	1 (3.7)	-	1 (2.1)	-	
		Medial	3 (3.1)	1 (3.7)	2 (7.4)	-	3 (6.4)	-	
		Lateral	1 (1)	1 (3.7)	-	-	1 (2.1)	-	
	Level	Proximal to the PSB	1 (1)	-	2 **(7.4)**	-	2 (4.3)	-	**0.038**
		At the level of the PSB	4 (4.1)	-	2 (7.4)	1 (1.1)	3 (6.4)	2 (3.6)	
		Distal to the PSB	2 (2.1)	-	2 (7.4)	1 (1.1)	2 (4.3)	-	
		Body	1 (1)	1 (3.7)	1 (3.7)	-	2 (4.3)	-	

	Pathology type	Irregularity	4 (4.1)	1 (3.7)	1 (3.7)	2 (2.3)	4 (8.5)	2 (3.6)	
		Enlargement	1 (1)	-	**2 (7.4)**	**-**	2 (4.3)	-	**0.038**
		Fluid-based hyperintensity	4 (4.1)	1 (3.7)	2 (7.4)	1 (1.1)	2 (4.3)	2 (3.6)	
		Adhesions	3 (3.1)	-	2 (7.4)	-	-	-	
		Chronic fibrosis	1 (1)	-	1 (3.7)	-	-	-	
